# Modeling *Dinophysis* in Western Andalucía using an autoregressive hidden Markov model

**DOI:** 10.1007/s10651-022-00534-7

**Published:** 2022-05-04

**Authors:** Jordan Aron, Paul S. Albert, Matthew O. Gribble

**Affiliations:** 1 Biostatistics Branch, Division of Cancer and Epidemiology, National Cancer Institute, Rockville, MD, USA; 2 Department of Epidemiology, University of Alabama at Birmingham School of Public Health, Birmingham, AL, USA

**Keywords:** Autoregressive, EM algorithm, Harmful algal bloom, Missing data, Toxins

## Abstract

*Dinophysis* spp. can produce diarrhetic shellfish toxins (DST) including okadaic acid and dinophysistoxins, and some strains can also produce non-diarrheic pectenotoxins. Although DSTs are of human health concern and have motivated environmental monitoring programs in many locations, these monitoring programs often have temporal data gaps (e.g., days without measurements). This paper presents a model for the historical time-series, on a daily basis, of DST-producing toxigenic *Dinophysis* in 8 monitored locations in western Andalucía over 2015–2020, incorporating measurements of algae counts and DST levels. We fitted a bivariate hidden Markov Model (HMM) incorporating an autoregressive correlation among the observed DST measurements to account for environmental persistence of DST. We then reconstruct the maximum-likelihood profile of algae presence in the water column at daily intervals using the Viterbi algorithm. Using historical monitoring data from Andalucía, the model estimated that potentially toxigenic *Dinophysis* algae is present at greater than or equal to 250 cells/L between < 1% and >10% of the year depending on the site and year. The historical time-series reconstruction enabled by this method may facilitate future investigations into temporal dynamics of toxigenic *Dinophysis* blooms.

## 1 Introduction

Following the identification of *Dinophysis fortii* as the causative agent of shellfish poisoning outbreaks in 1976 and 1977 in northeastern Japan, there has been interest in understanding the dinoflagellates of the genus *Dinophysis* (Yasumoto et al. 1978, 1980; FAO and WHO 2016). *Dinophysis* has been shown to be toxigenic in a variety of environments (Fux et al. 2011; Mafra et al. 2013; Gao et al. 2017), and a global distribution system for these toxins is generally recognized (FAO and WHO 2016) as they occur in a multitude of different habitats. To date, 10 species of *Dinophysis* have been shown to produce one or two major types of lipophilic toxins, okadaic acid (OA) and its dinophysistoxins derivatives, and pectenotoxins (Reguera et al. 2012, 2014). We collectively refer to the set OA, its derivatives, and pectenotoxins as diarrhetic shellfish toxins (DST). While all DST are known to be harmful, OA is particularly important to study due to the knowledge gap about its potential for understudied chronic disease associations (IOC et al. 2005) as well as its likely tumor promotion properties (Suganuma et al. 1988; Fujiki et al. 2018; Valdiglesias et al. 2013). The potential for low-dose chronic exposures of human populations to OA (along with pectenotoxins) is environmentally plausible because the toxins may persist in the water for extended periods of time (Blanco et al. 2018; Pizarro et al. 2009) and the toxin has been found in the absence of *Dinophysis* (Fernández et al. 2019).

In 1994 the autonomous government of Andalucía implemented a phytoplankton monitoring system (Fernández et al. 2019). Since the late 1990s, multiple species of *Dinophysis* (e.g., *D. acuminata complex, D. caudata, D. acuta, and D. fortii*) have been detected in most of the sampled areas (Fernández et al. 2019). Levels of toxins have been found in various species of edible shellfish (e.g. *Callista chione* and *Venus verrucose*), sometimes exceeding the legal limit of 160 *μ*g/kg (e.g. *Donax trunculus, Chamelea gallina, Mytilus galloprovincialis*, and *Cerastoderma edule*) (Fernández et al. 2019). The concentration of DST in a shellfish at a given time is a function of shellfish-specific features such as the rate of uptake, biotransformation, and elimination of each particular toxin (Reguera et al. 2014), but also reflects the environmental dynamics of toxigenic algae.

Previous efforts to model the dynamics of toxigenic *Dinophysis* have had limitations. Artificial neural networks have been applied to the coast of Huelva in Andalucía (Velo-Suárez and Estrada 2007); however, that modeling effort used the last 5 weekly *D. acuminata* concentrations as the only input variables to predict the upcoming week’s concentration. Kulawiak (2016) used GIS and advanced very-high-resolution radiometer data to detect algae blooms in the Baltic sea, but this did not leverage toxin measurements. To our knowledge, it has been an unaddressed modeling challenge to account for the fact that DST can persist in water for extended periods of time, while also giving interpretable model parameters. Hidden Markov Models (HMM) have proven to be an effective modeling tool (Zucchini and Macdonald 2009). In addition, they have specifically been used to study algal blooms in other contexts and provide a potential scaffold for an improved *Dinophysis* model. Rousseeuw et al. (2015) used a hybrid HMM to detect and understand the dynamics of phytoplankton blooms in France using data on nutrients and water characteristics, but lacking direct data on algae. In the freshwater harmful algal bloom modeling field, Jiang et al. (2016) employed a continuous HMM alongside principal component analysis of water quality parameters and nutrient to forecast microcystins. Kim et al. (2020) analyzed chlorophyll-a concentrations, a metric to understand eutrophication, in the Nakdong river of South Korea using a continuous HMM. All of these approaches model a multivariate outcome of water quality and chemical parameters using a HMM with an unknown number of states. Rousseeuw et al. (2015) and Jiang et al. (2016) reduce the dimension of the multivariate outcome by clustering and principal components, respectively. Kim et al. (2020) models the spatial distribution of chlorophyll-a conditional on the latent state. In our work, we use a 2-state HMM to directly infer whether there is algae in the water column over time. This is important because we want to reconstruct a daily assessment of this variable for health surveillance. Autoregressive HMMs on the other hand, were initially developed for speech recognition (Juang and Rabiner 1986, 1985). They have since been applied to various issues in recent years (Urban et al. 2020; Bartolucci et al. 2014; Shannon and Byrne 2010), but have not been used to model algae.

This paper presents a first-order autoregressive HMM approach to modeling potentially toxigenic *Dinophysis* in western Andalucía with the purpose of reconstructing the maximum-likelihood profile of whether algae were absent or present in the water column above a threshold count (e.g., ≥ 500 cells / L) at daily intervals using incomplete time-series historical data on both DST levels and algal counts. We model the presence/absence of algae in the water column by using algae cell counts from water column samples and DST measurements (in *μg* of OA equivalents per kg) from shellfish gathered from the regional government’s website. Then, using the estimated model parameters we can reconstruct indicators of algae in the water column at a daily interval, even when data is often missing. Since OA can remain in the water for extended periods of time, it is important that we allow for serial dependence in the model formulation. Specifically, we introduce autoregressive dependence in the observed DST measurements after accounting for the hidden Markov structure. The forward-backward algorithm needed to be adapted (Stanculescu et al. 2014) for computing E-step calculations in order to implement the EM algorithm for maximum-likelihood in this setting. We assume a first order autoregressive model as algae blooms erupts quickly and this assumption provides a useful framework over a longer time horizon to capture the quickly moving event. Section 2 presents an in-depth explanation of the model while Sect. 3 explains the estimation procedure, along with the adapted forward-backward algorithm that can account for both missing data and dependence on previously observed states. In Sect. 4 we talk about three simulations which compare estimation with different amounts of missing data. In the 5^th^ section we apply our model to the Andalucía data and discuss the estimated parameters and we consider our results in Sect. 6.

## 2 Methods

We consider a binary first order autoregressive HMM for the true algae state in the water column to model water sample algae counts and DST. We assume that the true water column algae state is binary as algae can either be absent or present in the water column. Let $S_{t}$, $X_{t}$ and $Y_{t}$ denote the daily true water column algae state, water sample algae count, and DST state, respectively, for day *t* where 1<t<T. The domain for these three variables is defined below. Also let $S=\left(S_{1},\ldots ,S_{T}\right)$, and similarly for **X** and **Y**. **S**, **X** and **Y** all have the same follow up with equally spaced daily observations.

We assume that algae in the water column can be modelled by a Markov chain, where $S_{t}$ is either 0 or 1 depending on if algae is absent or present in the water column. We define the notation *r*_*S_t_*_ to be the probability of starting in state $S_{t}$ at time *t* = 1 and $PS_{t-1}S_{t}$ to be the probability of transitioning from state $S_{t-1}$ to state $S_{t}$ at time *t*. Specifically, $S_{t}∼MC\left(p_{01},p_{10},r_{1}\right)$ where *p*_01_ denotes the probability of initiating water column algae over a day, *p*_10_ indicates the probability of ending the episode over a day, and *r*_1_ denotes the probability of being in the algae state at time *t* = 1. For use in calculations, we also define *p*_00_ as the probability of algae remaining out of the water column over a day, *p*_11_ as the probability of algae continuing to remain in the water column over a day, and *r*_0_ as the probability of not being in the algae state at time *t* = 0

To model the algae cell counts from the water sample, we chose a negative binomial as it can account for overdispersion in the count data. $X_{t}$ takes on the integer count of algal cells in a sample of water and is conditional on $S_{t}$. Thus the possible values of $X_{t}$ are $0\leq X_{t}\leq \infty$. Although $X_{t}$ is directly observed, it is also subject to measurement error. Due to the spatial heterogeneity of the algae and the water sampling technique used (Fernández et al. 2019), excess zeros are possible based on the specific latitude and longitude sampled. When $X_{t}$ = 0 we can’t be sure if the sample missed the algae or if algae is truly absent from the water column, however when $X_{t}$ > 0 we know for sure that algae is present in the water column. Reversing this, when $S_{t}$ = 0 then $X_{t}$ = 0 but when $S_{t}$ = 1 then it is possible but not necessary that $X_{t}$ be greater than 0. We chose to model the absence and presence of algae (quantified as above or below the 50 cells/L detection limit), however other thresholds can easily be chosen. Appendix A discusses the implications when two additional thresholds are considered. When algae is present in the water column, we model $X_{t}$ with a negative binomial distribution with mean *μ*_*a*_ and size *k*_*a*_ where $E\left[X_{t}|S_{t}=1\right]=\mu_{a}$ and $V\left[X_{t}|S_{t}=1\right]=\mu_{a}+\frac{\mu_{a}^{2}}{k_{a}}$. This relationship can be concisely summed up as:

(1) $$X_{t}=\{\begin{array}{ll} 0 & \text{if}\;S_{t}=0\\ NB\left(\mu_{a},\;k_{a}\right) & \text{if}\;S_{t}=1. \end{array}$$

We discretized the DST measurements in to four states as a large proportion of values are below the quantification limit and are non-normally distributed on all commonly considered transformed scales. Discrete measurements also help with the computational feasibility of the method. Therefore, let $Y_{t}^{*}$ represents the continuous toxin measurements and let $Y_{t}$ represent the discretized measurements as follows:

(2) $$Y_{t}=\{\begin{array}{ll} 0 & \text{if}\;Y_{t}^{*}\leq 40\mu g\;\text{of OA equ}/\text{kg}\\ 1 & \text{if}\;40<Y_{t}^{*}\leq 100\mu g\;\text{of OA equ}/\text{kg}\\ 2 & \text{if}\;100<Y_{t}^{*}\leq 160\mu g\;\text{of OA equ}/\text{kg}\\ 3 & \text{if}\;Y_{t}^{*}>160\mu g\;\text{of OA equ}/\text{kg} \end{array}$$

These specific cutoffs were chosen as the limit of detection is 40*μg* and fisheries must close at 160*μg* while 100*μg* lies halfway between the two other constraints. The DST states follow an ordinal logistic regression model with two regression parameters and three intercept parameters. The full model is,

(3) $$\text{logit}\left(P\left(Y_{t}\leq c\right)\right)=\alpha_{c}+\beta_{1}\times Y_{t-1}+\beta_{2}\times S_{t}$$

where 0≤c≤2, *α*_*c*_ is the intercept parameter, *β*_1_ is the regression parameter for the last toxin measurement, and *β*_2_ is the regression parameter for the current Markov chain state. The regression coefficients can be interpreted as: there is $e^{\beta_{1}}$ times the odds of $Y_{t}$ = *c* + 1 compared to $Y_{t}$ = *c* with each increase by one in *Y*_*t* − 1_ and when $S_{t}$ increases from 0 to 1 there is $e^{\beta_{2}}$ times odds of $Y_{t}$ = *c* + 1 compared to $Y_{t}$ = *c*. The dependence of $Y_{t}$ on $Y_{t-1}$ creates issues when $Y_{t-1}$ is missing, however these problems will be dealt with in Sect. 3.

The joint distribution of the latent water column algae state, the observed water sample algae count, and DST measurement can be calculated by multiplying the following three components: (1) the probability of being in a water column algae state, (2) conditional on the water column state, the probability of the algae cell count from the water sample, and (3) conditional on the water column state and the last DST state, the probability of the current DST state. The complete-data joint distribution can be written as:

(4) $$\begin{array}{l} f(S,X,Y)=r_{S_{1}}\prod_{t=2}^{T}p_{S_{t-1}S_{t}}\\ \;\times \prod_{t=1}^{T}NB{\left(X_{t}|\mu_{a},k_{a}\right)}^{S_{t}}\\ \;\times \prod_{t=1}^{T}P\left(Y_{t}|S_{t},Y_{t-1}\right). \end{array}$$

We assume that *Y*_0_ = 0 because most values are of $Y_{t}$ are zero. Additionally as a sensitivity analysis we ran our analysis where *Y*_0_ = 1, *Y*_0_ = 2, and *Y*_0_ = 3 and the results did not change. The estimation procedures are described in the next section. Parametric bootstrap standard errors were calculated by simulating data 500 times per site using the estimated parameters. Bootstrap standard errors were then calculated for each parameter by calculating the standard deviation of the 500 samples. Finally, we apply the Viterbi algorithm to reconstruct the highest likelihood hidden state path.

## 3 Estimation

We will now introduce the procedure for estimation assuming no missing data. Define the indicator variable *Z* such that

$$Z_{S_{t}}\left(S_{t}\right)=\{\begin{array}{ll} 0 & \text{if}\;S_{t}\neq s_{t}\\ 1 & \text{if}\;S_{t}=s_{t}. \end{array}$$

This indicator function is critical for later computations and can be used with variables other than $S_{t}$. We adopt the notation where $S_{t}$ refers to the random variable, while *s*_*t*_ refers to a possible value of the random variable $S_{t}$. We use this notation across all random variables. This indicator function is equal to 1 when the random variable of choice is equal to a specific realization of the random variable. We can then rewrite the complete-data joint distribution as:

(5) $$\begin{array}{l} f(S,X,Y)=\prod_{s_{1}=0}^{1}r_{s_{1}}^{Z_{s_{1}}\left(S_{1}\right)}\prod_{t=2}^{T}\prod_{s_{1}=0}^{1}\prod_{s_{2}=0}^{1}p_{s_{t-1}S_{t}}^{Z_{s_{t-1}}\left(S_{t-1}\right)Z_{s_{t}}\left(S_{t}\right)}\\ \;\times \prod_{t=1}^{T}NB{\left(X_{t}=x_{t}|\mu_{a},k_{a}\right)}^{S_{t}}\\ \;\times \prod_{t=1}^{T}\prod_{s_{t}=0}^{1}P{\left(Y_{t}=y_{t}|S_{t}=s_{t},Y_{t-1}=y_{t-1}\right)}^{Z_{s_{t}}\left(S_{t}\right)}. \end{array}$$

As **S** is not observable, to maximize this likelihood directly we would have to iterate over every possible value, thus calculating

(6) $$\sum_{s_{1}}^{k}\cdots \sum_{s_{T}}^{k}f\left(\left(s_{1},\ldots ,s_{T}\right),X,Y\right).$$

Maximizing this directly becomes intractable as *T* increases and as *T* = 2177 days, it is not feasible for our application. By using the expectation-maximization (EM) algorithm we can maximize this likelihood in a timely manner by alternating between an expectation and a maximization step, converging at the estimated parameters. The expectation step calculates the following complete-data log likelihood:

(7) $$\begin{array}{l} E[\log f(S,X,Y)|X,Y]=\sum_{s_{1}=0}^{1}E\left[Z_{s_{1}}\left(S_{1}\right)|X,Y\right]\log\left(r_{s_{1}}\right)\\ \;+\sum_{t=2}^{T}\sum_{s_{t-1}=0}^{1}\sum_{s_{t}=0}^{1}E\left[Z_{s_{t-1}}\left(S_{t-1}\right)Z_{s_{t}}\left(S_{t}\right)|X,Y\right]\log\left(p_{s_{t-1}s_{t}}\right)\\ \;+\sum_{t=1}^{T}E\left[S_{t}|X,Y\right]\log\left(NB\left(x_{t}|\mu_{a},k_{a}\right)\right)\\ \;+\sum_{t=1}^{T}\sum_{s_{t}=0}^{1}E\left[Z_{s_{t}}\left(S_{t}\right)|X,Y\right]\log\left(P\left(y_{t}|s_{t},y_{t-1}\right)\right), \end{array}$$

where the expectations can all be calculated using the forward-backward algorithm (Baum 1972).

### 3.1 Estimation with missing data

**Fig. 1:**
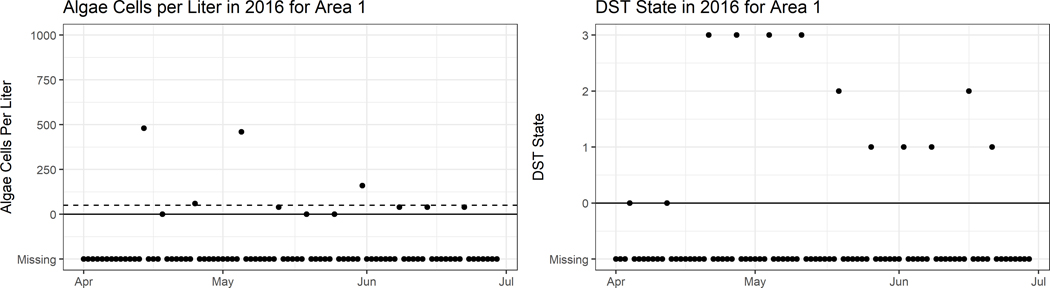
Sample of observations for a three month period in 2016 for area 1. The left panel shows algae count observations while the right shows DST state observations. Dots correspond to observations, while x’s signify a missing observation. The dashed line on the left shows the algae threshold of 50 cells/L

**Fig. 2:**
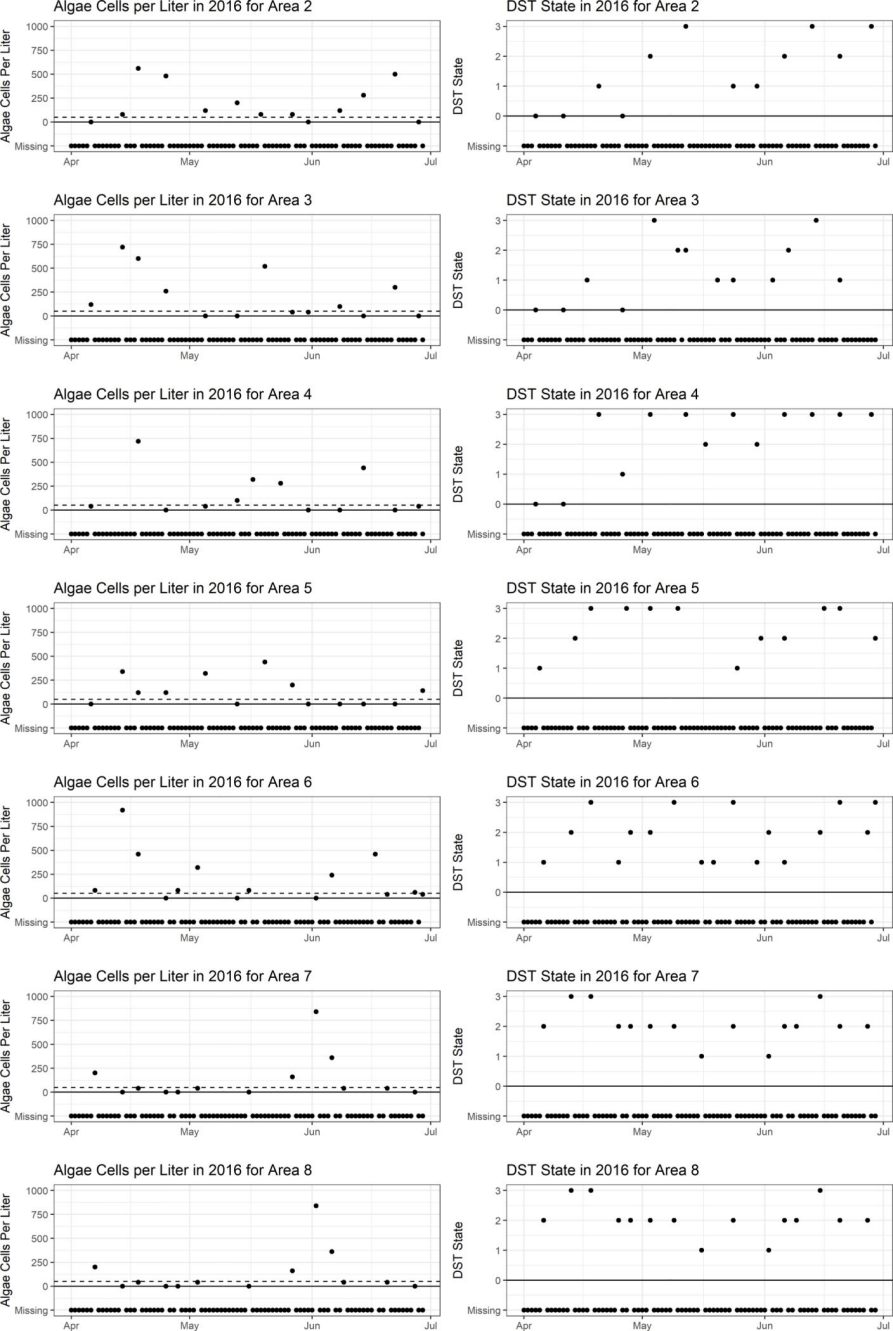
Decoded water column state ($S_{t}$) path using the Viterbi algorithm for 2016 in area 1. The line is the decoded path while the dots indicate absence and presence of observed algae. When algae counts are above 0 we know that $S_{t}$ must equal one, however when algae counts are 0 we cannot say anything about $S_{t}$. For the figure, this is why when we observe algae (indicated by a dot at 1) the path (the line) must pass through it, but when we do not observe algae (a dot at 0) the path may or may not pass through it

Often times some parts of the observable data are missing. As shown in Fig. 1 most observations are missing (other areas are shown in Fig. 2). This time frame was chosen as spring and summer are often when most algae blooms occur. In our example, when $X_{t}$ is missing we simply leave out the second line of the likelihood calculation, however when $Y_{t}$ is missing a more complicated method is required. As the current DST state depends on the last DST state, when $Y_{t}$ is missing we must account for it to calculate the probability of $Y_{t+1}$. By conditioning over all possible DST states for $Y_{t}$, we can calculate the probability of $Y_{t+1}$. The complete data joint distribution, accounting for missing data, can be written as

(8) $$\begin{array}{l} f(S,X,Y)=\prod_{s_{1}=0}^{1}r_{s_{1}}^{Z_{s_{1}}\left(S_{1}\right)}\prod_{t=2}^{T}\prod_{s_{t-1}=0}^{1}\prod_{s_{t}=0}^{1}p_{s_{t-1}s_{t}}^{Z_{s_{t-1}}\left(S_{t-1}\right)Z_{s_{t}}\left(S_{t}\right)}\\ \;\times \prod_{t=1}^{T}NB{\left(X_{t}=x_{t}|\mu_{a},k_{a}\right)}^{Z_{1}\left(S_{t}\right)}\\ \;\times \prod_{t=1}^{T}\prod_{s_{t}=0}^{1}\prod_{y_{t-1}=0}^{3}\prod_{y_{t}=0}^{3}P{\left(Y_{t}=y_{t}|S_{t}=s_{t},Y_{t-1}=y_{t-1}\right)}^{Z_{s_{t}}\left(S_{t}\right)Z_{y_{t-1}}\left(Y_{t-1}\right)Z_{y_{t}}\left(Y_{t}\right)}, \end{array}$$

where the complete-data log likelihood is now

(9) $$\begin{array}{l} E[\log f(S,X,Y)|X,Y]=\sum_{s_{1}=0}^{1}E\left[Z_{s_{1}}\left(S_{1}\right)|X,Y\right]\log\left(r_{s_{1}}\right)\\ \;+\sum_{t=2}^{T}\sum_{s_{t-1}=0}^{1}\sum_{s_{t}=0}^{1}E\left[Z_{s_{t-1}}\left(S_{t-1}\right)Z_{s_{t}}\left(S_{t}\right)|X,Y\right]\log\left(p_{s_{t-1}s_{t}}\right)\\ \;+\sum_{t=1}^{T}E\left[Z_{1}\left(S_{t}\right)|X,Y\right]\log\left(NB\left(x_{t}|\mu_{a},k_{a}\right)\right)\\ \;+\sum_{t=1}^{T}\sum_{s_{t}=0}^{1}\sum_{y_{t-1}=0}^{3}\sum_{y_{t}=0}^{3}\\ \;E\left[Z_{s_{t}}\left(S_{t}\right)Z_{y_{t-1}}\left(Y_{t-1}\right)Z_{y_{t}}\left(Y_{t}\right)|X,Y\right]\log\left(P\left(y_{t}|s_{t},y_{t-1}\right)\right). \end{array}$$

To account for the missing data and the dependency in the emissions distribution we use the adapted forward-backward algorithm from Stanculescu et al. (2014), however our application has a bivariate rather than univariate emissions distribution. For the maximization step, we maximize equation (9) given the E step calculations. The E step calculations are hard to calculate so we use the Forward-Backward algorithm described in the next section. We consider convergence to occur when the log likelihood increase between iterations is less than 0.01.

### 3.2 Forward-backward algorithm

To account for missing toxin values, we will keep track of every possible DST state value. Assume that $Y_{t}$ is missing. By calculating the probability of all possible DST states at time *t*, we can then calculate the probability of $Y_{t+1}$. We redefine the indicator variable *Z* to account for the scenario of missing data. Let

$$Z_{y_{t}}\left(Y_{t}\right)=\{\begin{array}{ll} 0 & \text{if}\;Y_{t}\neq y_{t}\\ 1 & \text{if}\;Y_{t}=y_{t}\;\text{or if}\;y_{t}\;\text{is missing}\;. \end{array}$$

The forward quantity is:

(10) $$\alpha_{s_{t}}(t,\omega )=P\left(X_{1}=x_{1},\ldots ,X_{t}=x_{t},Y_{1}=y_{1},\ldots ,Y_{t}=\omega ,S_{t}=s_{t}\right)\times Z_{y_{t}}(\omega ).$$

The indicator function, not present in the standard forward quantity, allows us to incorporate different possible values of $Y_{t}$ when $Y_{t}$ is missing. If $Y_{t}$ is observed the forward quantity is zero except when *y*_*t*_ = *ω*. However, when $Y_{t}$ is missing, *ω* corresponds to a possible DST state value at time *t*. This quantity is calculated recursively by

(11) $$\alpha_{s_{t}}(t,\omega )=\{\begin{array}{ll} r_{s_{1}}NB{\left(x_{1}|\mu_{a},k_{a}\right)}^{s_{1}}P\left(Y_{1}=\omega |s_{1},Y_{0}=0\right)\times Z_{Y_{1}}(\omega ) & \text{if}\;t=1\\ \sum_{s_{t-1}=0}^{1}\sum_{\omega_{0}=0}^{3}\alpha_{s_{t-1}}\left(t-1,\omega_{0}\right)p_{s_{t-1}s_{t}}NB{\left(x_{t}|\mu_{a},k_{a}\right)}^{s_{t}}P\left(Y_{t}=\omega |s_{t},Y_{t-1}=\omega_{0}\right)\times Z_{y_{t}}(\omega ) & \text{if}\;t>1. \end{array}$$

The backward quantity is defined as

(12) $$\beta_{s_{t}}(t,\omega )=P\left(X_{t+1}=x_{t+1},\ldots ,X_{T}=x_{T},Y_{t+1}=y_{t+1},\ldots ,Y_{T}=y_{T}|S_{t}=s_{t},Y_{t}=\omega \right)\;\;\times Z_{y_{t}}(\omega ),$$

where, similarly to the forward quantity, if $Y_{t}$ is observed the backward quantity is zero except when *y*_*t*_ = *ω* and if $Y_{t}$ is missing *ω* corresponds to a possible DST state value at time *t*. It is also calculated recursively:

(13) $$\beta_{s_{t}}(t,\omega )=\{\begin{array}{ll} 1 & \text{if}\;t=T\\ \sum_{s_{t+1}=0}^{1}\sum_{\omega_{0}=0}^{3}p_{s_{t}s_{t+1}}NB{\left(x_{t+1}|\mu_{a},k_{a}\right)}^{s_{t+1}}P(Y_{t+1}=\omega_{0}|s_{t+1},Y_{t}=\omega )\beta_{s_{t+1}}\left(t+1,\omega_{0}\right)\times Z_{y_{t}}(\omega ) & \text{if}\;t<T. \end{array}$$

### 3.3 Calculating expectations

The expectations from the complete-data log likelihood are calculated as follows:

(14) $$E\left[Z_{s_{t}}\left(S_{t}\right)|X,Y\right]=P\left(S_{t}=s_{t}|X,Y\right)=\frac{\sum_{\omega =0}^{3}\alpha_{s_{t}}(t,\omega )\beta_{s_{t}}(t,\omega )}{P(X,Y)}$$

(15) $$\begin{array}{l} E\left[Z_{S_{t-1}}\left(S_{t-1}\right)Z_{S_{t}}\left(S_{t}\right)|X,Y\right]\\ \;=\frac{P\left(S_{t-1}=s_{t-1},S_{t}=S_{t},X,Y\right)}{P(X,Y)}\\ \;=\frac{\sum_{\omega_{1}=0}^{3}\sum_{\omega_{2}=0}^{3}\alpha_{s_{t-1}}\left(t-1,\omega_{1}\right)p_{s_{t-1}s_{t}}g{\left(x_{t}|\mu_{a},k_{a}\right)}^{S_{t}}P\left(Y_{t}=\omega_{2}|s_{t},Y_{t-1}=\omega_{1}\right)\beta_{s_{t}}\left(t,\omega_{2}\right)}{P(X,Y)} \end{array}$$

(16) $$\begin{array}{l} E\left[Z_{S_{1}}\left(S_{t}\right)Z_{y_{t-1}}\left(Y_{t-1}\right)Z_{y_{t}}\left(Y_{t}\right)|X,Y\right]\\ \;=\frac{P\left(S_{t}=s_{1},Y_{t-1}=y_{t-1},Y_{t}=y_{t},X,Y\right)}{P(X,Y)}\\ \;=\sum_{s_{0}}\frac{P\left(S_{t}=s_{1},Y_{t-1}=y_{t-1},Y_{t}=y_{t},X,Y|S_{t-1}=s_{0}\right)}{P(X,Y)}\\ \;=\sum_{s_{0}}\frac{\alpha_{s_{0}}\left(t-1,y_{t-1}\right)p_{s_{0}s_{1}}g{\left(x_{t}|\mu_{a},k_{a}\right)}^{s_{1}}P\left(y_{t}|s_{1},y_{t-1}\right)\beta_{s_{1}}\left(t,\;y_{t}\right)}{P(X,Y)} \end{array}$$

(17) $$P(X,Y)=\sum_{s_{T}=0}^{1}\sum_{\omega =0}^{3}\alpha_{s_{T}}(T,\;\omega )$$

In equation 15 and 16 the function $g\left(x_{t}|\mu_{a}k_{a}\right)$ is the probability density function of negative binomial distribution with parameters *μ*_*a*_ and *k*_*a*_, calculating the probability of *x*_*t*_.

## 4 Simulation

We examine the performance of our proposed method by analyzing three simulations with varying amounts of missing data. We simulated data sets with no missing data, one-third of the data missing, and 85% of the data missing. For each category, 500 data sets were generated with the same follow up length as the data (2177 days). The simulated data structure corresponds to the application presented in our application section. These three amounts of missing data were chosen as they account for a wide variety of scenarios while also testing this specific application, which has (depending on the site) at most 83% of the data missing. By varying the level of missingness, we can measure how well our method preforms at recovering the true parameters with different levels of information available. It should also be noted that this is especially important to test for the DST measurements. When there is no missing data the estimation for the DST is straightforward, however with missing data the adapted forward backward algorithm explained in the estimation section is needed. With more missing data there are longer times between observations, meaning more reliance on the proposed adaption to account for $Y_{t}$ when calculating the probability of $Y_{t+1}$.

For the simulations that have no missing data, the estimates are extremely accurate with minimal standard errors. Table 1 contains the simulation estimates for the three different levels of missing data, and shows that our method is extremely accurate at recovering the true parameters regardless of missing data. Additional computation time is required when our method encounters missing data as all possible values of the last DST state are iterated over. Thus, the time needed for our method scales linearly with the amount of missing data.

Even with most of the data missing, our method accurately estimates the parameters. However, as more data is missing, the standard errors increase. While this increase is quite small when one-third of the data is missing, it is much larger when 85% of the data is missing. The standard errors for the 33% missing simulation roughly double when compared to the 0% missing simulation, however the standard errors increase by a factor ranging from 9 to 37 when comparing the 85% and the 0% missing simulations. This can most easily be seen in the third row of Table 1 for *μ*_*a*_. The estimate itself is accurate across the three levels, however the standard error when 85% of the data is missing is extremely large, even compared to the standard error when 33% of the data is missing. Despite this high variability, there is no relationship between the initial and estimated value in the simulations.

## 5 Application

### 5.1 Dataset description

We illustrate our method on data gathered from the regional government of Andalucía’s website (Zonas de producción. (n.d.) xxxx). The Andalucían government established a phytoplankton toxin monitoring program for shellfish in 1994 to help deal with the recurrent blooms of *Dinophysis* that are linked to DSP outbreaks (Bouza and Aboal 2008). We used data on toxin levels, measured in *μg* of OA equivalent/kg, sampled from the bivalve *Donax trunculus* in the time frame from January 2015 to December 2020. The follow-up length is 2,177 days. Toxin levels were calculated as specified by Yasumoto et al. (1984) and liquid chromatography-tandem mass spectrometry was used as the chemical analysis technique (Fernández et al.2019).Water column samples used to calculate algae cell counts were gathered using a 10-meter-long weighted plastic hose. 25mL water samples from sedimentation chambers were then used to extrapolate the number of cells/L (Velo-Suárez and Estrada 2007). Data from eight geographical sites (areas 1, 2, 3, 4, 5, 6, 7, and 8) were analyzed separately. Areas 7 and 8 were recorded as a single area until May 2018 and were then split into distinct areas; we analyzed each site in a separate model. Table 2 contains some summary statistics about the data.

### 5.2 Results

**Fig. 3:**
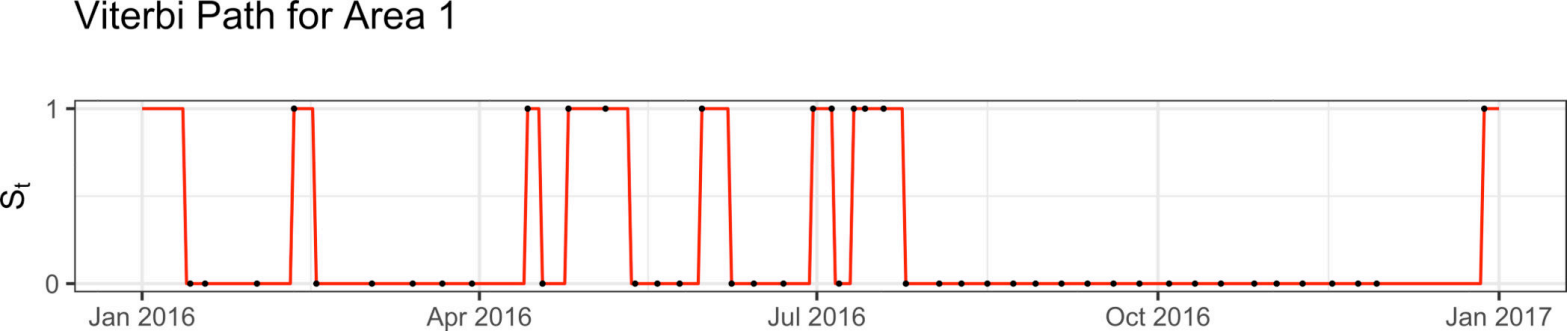
Proportion of predicted days with algae presence in the Viterbi path across all areas and years

**Fig. 4:**
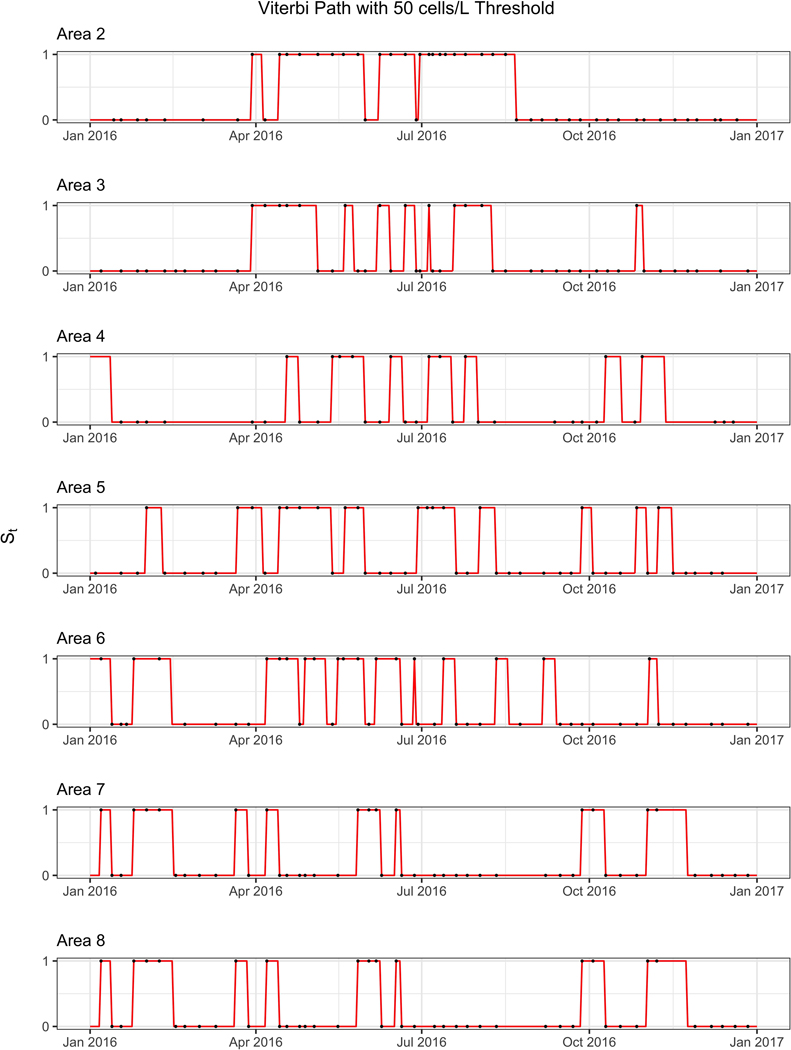
Sample of observations for a three month period in 2016 for area 2–8. Panels on the left shows algae count observations while panels on the right show DST state observations while areas are grouped by row. Dots correspond to observations, while x’s signify a missing observation. The dashed line on the left shows the algae threshold of 50 cells/L

**Fig. 5:**
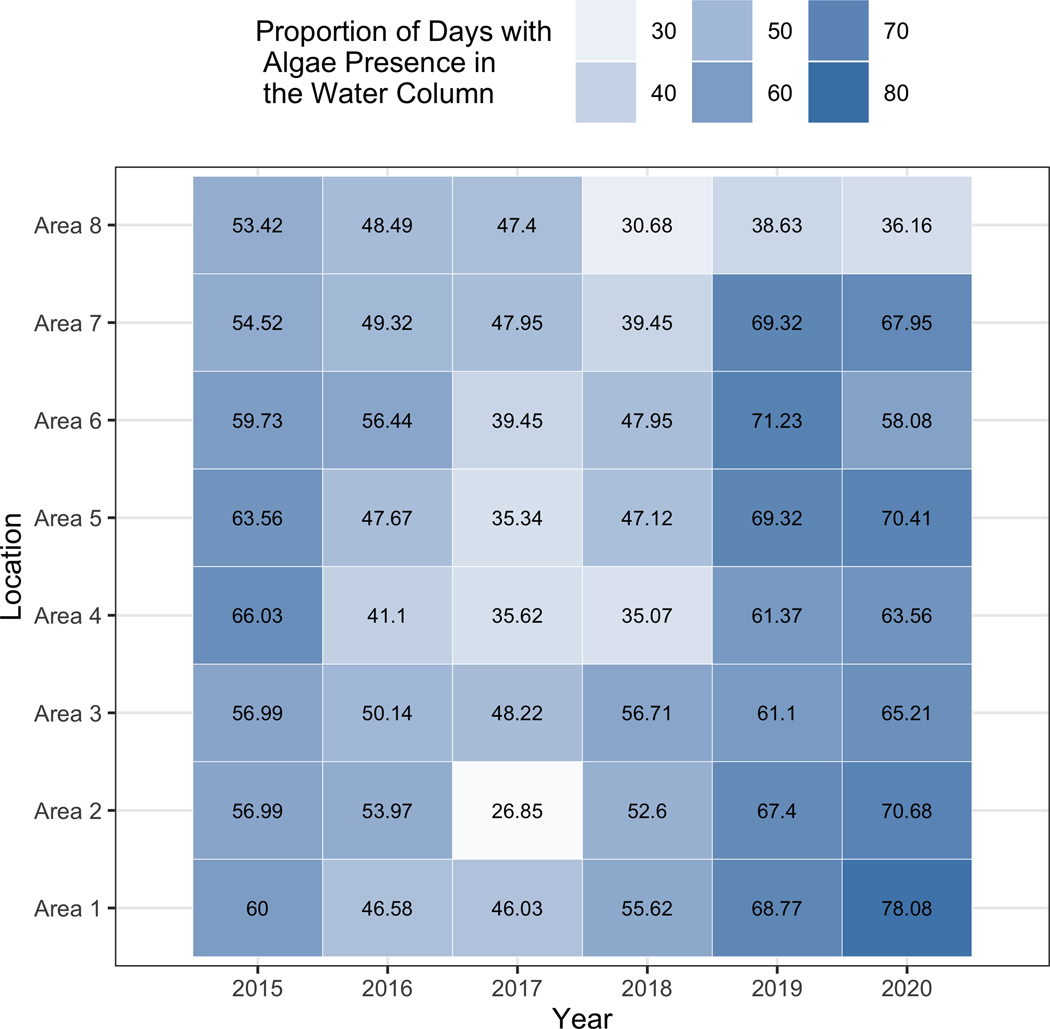
Decoded water column state ($S_{t}$) path using the Viterbi algorithm for 2016 for areas 2–8. The line is the decoded path while the dots indicate absence and presence of observed algae. When algae counts are above the threshold of 50 cells/L, indicated with a black dot at 1, $S_{t}$ must equal one

Our HMM has two states representing the presence or absence of potentially toxigenic *Dinophysis* algae in the water column at a concentration exceeding a threshold (e.g., ≥ 500 cells/L). Both the initiation (*p*_01_)and termination (*p*_10_) probabilities are low as can be seen in Table 2, indicating a tendency for algae to stay in or remain out of the water column for a number of days (corresponding to the 1 or 0 state of the HMM). Despite the minor differences between each of the different sites, there is broad homogeneity among the sites with the initiation probabilities being slightly lower than 10% and most termination probabilities hovering just above 10%. Within each area, initiation probabilities were lower than their corresponding termination probabilities. Although the state path of the hidden Markov model is unobserved, it is an important metric to recover as it can be useful in determining long-term changes in algae and can have implications for the effects of climate change. We reconstructed the hidden state paths using the Viterbi algorithm, producing the path with the highest likelihood. Figure 3 shows a visualization of the Viterbi path for area 1 in 2016 (other areas are shown in Fig. 4). Using the Viterbi path we can then calculate different summary statistics for algae presence/absence across time. As shown in Fig. 5, distinct trends can be seen within each year and across years. For instance, for areas 1–7 the earlier and later years have a higher proportion of algae in the water column when compared to the middle years. The proportion of days with algae was estimated to be at least 54.52 % and 61.1% for 2015 and 2019, while in 2017 it was estimated to be at most 48.22%.

As noted previously, we modeled the algae from the water sampled with a negative binomial model when $S_{t}$ = 1, and assumed that there cannot be any algae in the water sample when $S_{t}$ = 0. When there is algae in the water sample we can assume that $S_{t}$ = 1 because otherwise it would not be possible for there to be algae present. On the contrary, we cannot draw any conclusions when there is not algae in the water sample. This is the case because the algae cell count from the water sample is serving as an observable representative of algae in the water column with measurement error. Because the algae is not distributed evenly by either latitude, longitude, or depth, the water sample may not accurately capture whether algae is in the water column. As noted in the methods section, for this application we consider algae to be present in the water sample when it can be detected (a threshold of 50 cells per liter). We examine the consequences of higher thresholds in appendix A. Areas 3 and 5 have a mean parameter (*μ*_*a*_) around 230–240, while areas 1, 2, 4, and 6 have a higher mean parameter ranging from 265 to 290. Area 8 has a larger mean parameter of 320, and area 7 has a significantly larger mean around 440. Barring area 7, the size parameter (*k*_*a*_ in Eq. 1) is above 1 indicating that the negative binomial model is essential to help account for over-dispersion. As area 7 has the largest mean parameter and smallest size parameter (see expression 1), this leads to larger, more variable predictions for area 7.

The continuous DST measurements were discretized to form four (0 to 3) DST states, which follow an ordinal logistic model. The continuous measurements are highly skewed as the limit of quantification is 40 *μ*g of OA equ/kg. Binning the continuous measurements less than 40 *μ*g of OA equ/kg reduced the number of distributional assumptions. Unlike the algae cell count from the water sample that only depended on the current Markov chain state, we assume that the DST states are dependent on the current Markov chain state as well as the last DST state. This additional dependency is necessary in the emission distribution as major components of DST have been found to be very stable in the water column after a *Dinophysis* bloom (Blanco et al. 2018; Pizarro et al. 2009). This dependency cannot be estimated using the standard forward-backward algorithm as the standard HMM assumes that the observed states are all conditionally independent given the current latent state. In our model, the current observed state is dependent on the previous observed state and the current latent state, violating this assumption. Instead, by using procedures developed for autoregressive HMMs (Stanculescu et al. 2014), we can incorporate this dependency into the estimation procedure. We believe that a first order autoregressive model is applicable as algae blooms are rapid events. By having a shorter time dependency we are better able to model these events.

Our ordinal logistic model has five parameters: *β*_1_ is the effect of the DST state at time *t* − 1, *β*_2_ is the effect of the current Markov state, and *α*_0_, *α*_1_, and *α*_2_ are the intercept parameters. The effect of the last DST state is additive in relation to the log odds of the probability of the current DST state such that the effect of the last DST state is *β*_1_ when $Y_{t-1}=1,2\times \beta_{1}$ when $Y_{t-1}=2$, and 3 × *β*_1_ when $Y_{t-1}=3$. Table 3 contains all parameter estimates for the eight different areas along with their standard errors.

Despite the somewhat high standard errors for the regression coefficients, the probabilities themselves have a low standard error. The probabilities and standard errors for area 1, along with the other areas, are shown in Table 4. Interestingly, we can see the difference in predictive power between $S_{t}$ and $Y_{t}$ by looking this table. For each area, the difference between the left and right halves of the table is not nearly as drastic as the difference between the rows, indicating that the autoregressive effect on $Y_{t}$ is indispensable to this model.

## 6 Discussion

In this paper we have focused on the historical reconstruction of an incomplete time-series by developing a model that recreates the most likely pattern of *Dinophysis* spp. algae occurrence at each of the eight different sites on a daily timescale, using a HMM with extensions to account for challenges inherent to the data. DST measurements were highly autocorrelated, even after accounting for the hidden states of the HMM, violating one of the standard assumptions of HMMs (Rabiner and Juang 1986). However, by using an autoregressive HMM we are able to model this. The sampling frequency of the monitoring program resulted in large amounts of missing data (at most 83%). Furthermore, the distribution of DST was skewed and left-censored at the assay limits of quantification, 40*μg* of OA equivalents per kg (Zonas de producción.(n.d.) xxxx). We addressed these challenges with an advanced HMM that included a bivariate emissions distribution with a negative binomial distribution for algae counts and ordinal autoregressive model for the serial DST measurements. We showed with simulations that the approach accurately estimated the parameters even with extensive missing measurements.

This paper presents a modified forward-backward algorithm in an EM context from Stanculescu et al. (2014) with an additional observed variable applied to data from a phytoplankton toxin monitoring program in western Andalucía. This generalized form allows us to estimate a model with both dependence in the emissions distribution and missing data. In our application, DST states are dependent on both the current Markov state as well as the last DST state. The proposed method works by keeping track of all possible DST states (when the DST state is missing) in the forward-backward algorithm. We can then condition on and sum over the most recent DST state to calculate the probability of the current DST state. Although this does lead to additional computation complexity, the time needed scales linearly with the amount of missing data and is still feasible when nearly all of the data is missing.

We applied this method to 2,177 days of algae water samples and DST data from eight geographical sites with dates ranging from January 2015 to December 2020. Despite the long stretch of time covered in the study, most days had no recorded data. Although the data available varied by site, it ranged between 377 (17%) and 524 (24%) days with recorded measurements out of the total 2,177 days. Although HMMs have not been applied to this problem in this area before, our application of this method shows that HMMs are capable of modeling complex processes that don’t necessarily conform to the standard assumptions in the presence of large amount of missing data. Running our method on western Andalucía phytoplankton monitoring data we see that accounting for the last DST state requires the additional complexity of an autoregressive HMM.

One of the major advantages from our method is that we are able to reconstruct paths of the latent variable on a daily interval using historical time-series data in the presence of intermittent measurements and measurement error. Rather than forecast the future, our method focuses on predicting whether algae were absent or present in the water column for every day in our data set. This is useful when trying to identify long term algae trends for the different areas across time as well as for health surveillance. The Viterbi algorithm is ideal for computing estimates of the entire sequence of latent states. These sequences can later be used in downstream analyses that examine the relationship between toxicity and diseased risk. By aggregating these sequences, termed Viterbi paths, we are able to identify long term trends across years.

The proposed hidden Markov model makes a number of parametric assumptions including that the unobserved states follow a first-order Markov model and that the observed DST data follow a first-order autoregressive process after accounting for the HMM structure. We believe that these are reasonable assumptions since DST remains in the water for extended periods of time and algae blooms are rapid events. Latent state estimation should not be sensitive to small departures from these underlying assumptions. Therefore, we presume that the AR(1)-HMM framework adequately describes the biological process.

In the future, our method can be applied to other types of monitoring program data as well. Because most monitoring program data contains missing values, accounting for the temporal autocorrelation that is often present is not straightforward. Our method can adequately handle both complications simultaneously while also creating historical time-series reconstructions. Using our method, we are also able to relate two separate processes together while we impute the maximum-likelihood profile of the variable of interest.

## Supplementary Material

Appendix

## Figures and Tables

**Table 1:** Comparison of truth and estimated parameter from three different simulations with 0%, 33%, and 85% missing data

	Truth	0% Missing	33% Missing	85% Missing
*p*_01_	0.40	0.40 (0.001)	0.40 (0.001)	0.39 (0.009)
*p*_10_	0.20	0.20 (< 1e-04)	0.20 (< 1e-04)	0.20 (0.005)
*μ_a_*	64.00	63.81 (11.059)	64.46 (16.15)	65.63 (102.612)
*k_a_*	0.25	0.25 (< 1e-04)	0.25 (< 1e-04)	0.27 (0.003)
*β*_1_	1.00	1.00 (0.002)	1.00 (0.004)	1.00 (0.074)
*β*_2_	3.00	3.00 (0.049)	3.00 (0.097)	3.06 (1.717)
*α*_0_	3.00	3.00 (0.043)	2.98 (0.09)	2.97 (1.484)
*α*_1_	4.00	4.00 (0.054)	3.98 (0.111)	4.01 (1.824)
*α*_2_	5.00	5.00 (0.063)	4.98 (0.131)	5.01 (2.192)

Standard errors are in parenthesis

**Table 2:** Summary of basic information about the collected algae count and DST state data. DST continuous measurements were discretized into four states: 0 (≤ 40*μg* of OA equ/kg), 1 (40 ≤ 100*μg* of OA equ/kg), 2 (100 ≤ 160*μg* of OA equ/kg), and 3 (≥ 160*μg* of OA equ/kg)

	Area 1	Area 2	Area 3	Area 4	Area 5	Area 6	Area 7	Area 8
Average algae count	129.96	129.36	104.29	136.09	113.19	125.71	196.17	124.16
Average DST state	1.48	1.26	1.27	1.74	1.49	1.33	1.31	1.27
Days of followup	2177	2177	2177	2177	2177	2177	2177	2177
Days with observed data	524	506	485	377	463	474	483	430
Percentage of missing data	75.9%	76.8%	77.7%	82.7%	78.7%	78.2%	77.8%	80.2%
Days of <50 algae cells/L	159	162	168	119	156	153	157	144
Days of >50 algae cells/L	116	118	121	101	120	120	117	82
Days of 0 DST	63	83	79	44	59	83	84	86
Days of 1 DST	104	102	113	84	97	100	97	100
Days of 2 DST	95	95	95	98	107	96	90	88
Days of 3 DST	62	35	35	91	54	48	47	42

**Table 3:** Markov transition probabilities for algae presence/absence in the water column, mean and size negative binomial parameters for water sample algae count, and ordinal logistic regression coefficients for DST state

	*p*_01_	*p*_10_	*μ_a_*	*k_a_*	*β*_1_	*β*_2_	*α*_0_	*α*_1_	*α*_2_
Area 1	0.05 (0.01)	0.07 (0.02)	287.64 (25.44)	1.16 (0.22)	4.89 (0.27)	1.10 (0.29)	2.37 (0.22)	8.04 (0.49)	12.72 (0.78)
Area 2	0.10 (0.02)	0.15 (0.04)	286.13 (25.44)	1.10 (0.20)	4.93 (0.30)	0.84 (0.41)	2.67 (0.28)	8.01 (0.58)	12.82 (0.87)
Area 3	0.10 (0.02)	0.14 (0.04)	229.46 (16.18)	1.55 (0.28)	4.88 (0.33)	1.10 (0.39)	2.62 (0.29)	8.14 (0.64)	12.86 (0.95)
Area 4	0.07 (0.02)	0.11 (0.03)	280.99 (26.49)	1.10 (0.20)	5.61 (0.47)	1.51 (0.54)	2.85 (0.43)	9.16 (0.92)	14.40 (1.37)
Area 5	0.08 (0.02)	0.11 (0.03)	242.41 (20.08)	1.20 (0.23)	5.34 (0.36)	1.12 (0.44)	2.77 (0.35)	8.61 (0.69)	13.86 (1.06)
Area 6	0.11 (0.03)	0.15 (0.04)	266.43 (23.72)	1.14 (0.20)	4.87 (0.47)	1.42 (0.57)	2.83 (0.46)	8.18 (0.94)	12.99 (1.42)
Area 7	0.07 (0.02)	0.11 (0.04)	438.78 (49.94)	0.76 (0.15)	5.31 (0.38)	1.13 (0.45)	2.94 (0.34)	8.73 (0.72)	13.89 (1.10)
Area 8	0.06 (0.01)	0.10 (0.03)	320.39 (28.35)	1.14 (0.21)	5.30 (0.36)	1.57 (0.39)	2.96 (0.30)	8.81 (0.66)	14.15 (1.04)

*β*_1_ represents the effect of the DST at time *t* − 1, *β*_2_ represents the effect of the Markov state at time *t*, while *α*_*c*_ is the intercept coefficient. Standard errors are in parenthesis

**Table 4:** Probability of being in each DST state for every area given the last DST state and the current Markov state

	$S_{t}=0$				$S_{t}=1$			
	$Y_{t}=0$	$Y_{t}=1$	$Y_{t}=2$	$Y_{t}=3$	$Y_{t}=0$	$Y_{t}=1$	$Y_{t}=2$	$Y_{t}=3$
Area 1								
$Y_{t-1}=0$	0.91 (3e-04)	0.09 (3e-04)	0.00 (< 1e-4)	0.00 (< 1e-4)	0.78 (0.002)	0.22 (0.002)	0.00 (< 1e-4)	0.00 (< 1e-4)
$Y_{t-1}=1$	0.07 (1e-04)	0.88 (3e-04)	0.04 (1e-04)	0.00 (< 1e-4)	0.03 (1e-04)	0.86 (5e-04)	0.11 (5e-04)	0.00 (< 1e-4)
$Y_{t-1}=2$	0.00 (< 1e-4)	0.15 (5e-04)	0.80 (9e-04)	0.05 (2e-04)	0.00 (< 1e-4)	0.06 (2e-04)	0.81 (0.001)	0.14 (8e-04)
$Y_{t-1}=3$	0.00 (< 1e-4).	0.00 (< 1e-4)	0.12 (4e-04)	0.87 (4e-04)	0.00 (< 1e-4).	0.00 (< 1e-4)	0.05 (1e-04)	0.95 (1e-04)
Area 2								
$Y_{t-1}=0$	0.94 (3e-04)	0.06 (3e-04)	0.00 (< 1e-4)	0.00 (< 1e-4)	0.86 (0.001)	0.14 (0.001)	0.00 (< 1e-4)	0.00 (< 1e-4)
$Y_{t-1}=1$	0.09 (2e-04)	0.86 (4e-04)	0.04 (1e-04)	0.00 (< 1e-4)	0.04 (2e-04)	0.86 (4e-04)	0.10 (5e-04)	0.00 (< 1e-4)
$Y_{t-1}=2$	0.00 (< 1e-4)	0.13 (5e-04)	0.81 (7e-04)	0.05 (2e-04)	0.00 (< 1e-4)	0.06 (4e-04)	0.83 (7e-04)	0.11 (7e-04)
$Y_{t-1}=3$	0.00 (< 1e-4)	0.00 (< 1e-4)	0.12 (5e-04)	0.88 (5e-04)	0.00 (< 1e-4)	0.00 (< 1e-4)	0.06 (3e-04)	0.94 (3e-04)
Area 3								
$Y_{t-1}=0$	0.93 (3e-04)	0.07 (3e-04)	0.00 (< 1e-4)	0.00 (< 1e-4)	0.82 (0.0013)	0.18 (0.0013)	0.00 (< 1e-4)	0.00 (< 1e-4)
$Y_{t-1}=1$	0.09 (2e-04)	0.87 (4e-04)	0.04 (1e-04)	0.00 (< 1e-4)	0.03 (1e-04)	0.86 (5e-04)	0.10 (5e-04)	0.00 (< 1e-4)
$Y_{t-1}=2$	0.00 (< 1e-4)	0.17 (7e-04)	0.79 (9e-04)	0.04 (2e-04)	0.00 (< 1e-4)	0.06 (4e-04)	0.82 (8e-04)	0.12 (7e-04)
$Y_{t-1}=3$	0.00 (< 1e-4)	0.00 (< 1e-4)	0.14 (8e-04)	0.86 (8e-04)	0.00 (< 1e-4)	0.00 (< 1e-4)	0.05 (3e-04)	0.95 (3e-04)
Area 4								
$Y_{t-1}=0$	0.95 (3e-04)	0.05 (3e-04)	0.00 (< 1e-4)	0.00 (< 1e-4)	0.79 (0.003)	0.21 (0.003)	0.00 (< 1e-4)	0.00 (< 1e-4)
$Y_{t-1}=1$	0.06 (1e-04)	0.91 (3e-04)	0.03 (1e-04)	0.00 (< 1e-4)	0.01 (< 1e-4)	0.87 (7e-04)	0.11 (8e-04)	0.00 (< 1e-4)
$Y_{t-1}=2$	0.00 (< 1e-4)	0.11 (4e-04)	0.85 (7e-04)	0.04 (2e-04)	0.00 (< 1e-4)	0.03 (1e-04)	0.81 (0.0012)	0.16 (0.0014)
$Y_{t-1}=3$	0.00 (< 1e-4)	0.00 (< 1e-4)	0.08 (2e-04)	0.92 (2e-04)	0.00 (< 1e-4)	0.00 (< 1e-4)	0.02 (1e-04)	0.98 (1e-04)
Area 5								
$Y_{t-1}=0$	0.94 (3e-04)	0.06 (3e-04)	0.00 (< 1e-4)	0.00 (< 1e-4)	0.84 (0.0016)	0.16 (0.0016)	0.00 (< 1e-4)	0.00 (< 1e-4)
$Y_{t-1}=1$	0.07 (2e-04)	0.89 (3e-04)	0.04 (1e-04)	0.00 (< 1e-4)	0.02 (1e-04)	0.87 (5e-04)	0.10 (6e-04)	0.00 (< 1e-4)
$Y_{t-1}=2$	0.00 (< 1e-4)	0.11 (3e-04)	0.85 (6e-04)	0.04 (2e-04)	0.00 (< 1e-4)	0.04 (2e-04)	0.85 (7e-04)	0.11 (7e-04)
$Y_{t-1}=3$	0.00 (< 1e-4)	0.00 (< 1e-4)	0.10 (3e-04)	0.90 (3e-04)	0.00 (< 1e-4)	0.00 (< 1e-4)	0.04 (1e-04)	0.96 (1e-04)
Area 6								
$Y_{t-1}=0$	0.94 (3e-04)	0.06 (3e-04)	0.00 (< 1e-4)	0.00 (< 1e-4)	0.80 (0.0017)	0.19 (0.0017)	0.00 (< 1e-4)	0.00 (< 1e-4)
$Y_{t-1}=1$	0.12 (3e-04)	0.85 (6e-04)	0.03 (2e-04)	0.00 (< 1e-4)	0.03 (1e-04)	0.84 (7e-04)	0.13 (8e-04)	0.00 (< 1e-4)
$Y_{t-1}=2$	0.00 (< 1e-4)	0.17 (7e-04)	0.79 (9e-04)	0.04 (2e-04)	0.00 (< 1e-4)	0.05 (3e-04)	0.81 (9e-04)	0.14 (9e-04)
$Y_{t-1}=3$	0.00 (< 1e-4)	0.00 (< 1e-4)	0.16 (9e-04)	0.83 (9e-04)	0.00 (< 1e-4)	0.00 (< 1e-4)	0.05 (3e-04)	0.95 (3e-04)
Area 7								
$Y_{t-1}=0$	0.95 (2e-04)	0.05 (2e-04)	0.00 (< 1e-4)	0.00 (< 1e-4)	0.86 (0.0011)	0.14 (0.0011)	0.00 (< 1e-4)	0.00 (< 1e-4)
$Y_{t-1}=1$	0.09 (2e-04)	0.88 (3e-04)	0.03 (1e-04)	0.00 (< 1e-4)	0.03 (1e-04)	0.88 (4e-04)	0.09 (5e-04)	0.00 (< 1e-4)
$Y_{t-1}=2$	0.00 (< 1e-4)	0.13 (5e-04)	0.83 (7e-04)	0.04 (2e-04)	0.00 (< 1e-4)	0.05 (3e-04)	0.85 (7e-04)	0.11 (7e-04)
$Y_{t-1}=3$	0.00 (< 1e-4)	0.00 (< 1e-4)	0.11 (4e-04)	0.89 (4e-04)	0.00 (< 1e-4)	0.00 (< 1e-4)	0.04 (2e-04)	0.96 (2e-04)
Area 8								
$Y_{t-1}=0$	0.95 (2e-04)	0.05 (2e-04)	0.00 (< 1e-4)	0.00 (< 1e-4)	0.80 (0.0018)	0.20 (0.0018)	0.00 (< 1e-4)	0.00 (< 1e-4)
$Y_{t-1}=1$	0.09 (2e-04)	0.88 (4e-04)	0.03 (1e-04)	0.00 (< 1e-4)	0.02 (1e-04)	0.85 (6e-04)	0.13 (7e-04)	0.00 (< 1e-4)
$Y_{t-1}=2$	0.00 (< 1e-4)	0.14 (5e-04)	0.83 (7e-04)	0.03 (1e-04)	0.00 (< 1e-4)	0.03 (1e-04)	0.84 (8e-04)	0.12 (7e-04)
$Y_{t-1}=3$	0.00 (< 1e-4)	0.00 (< 1e-4)	0.15 (7e-04)	0.85 (7e-04)	0.00 (< 1e-4)	0.00 (< 1e-4)	0.03 (1e-04)	0.97 (1e-04)

Standard errors are included in parenthesis

**Fig. 6:**
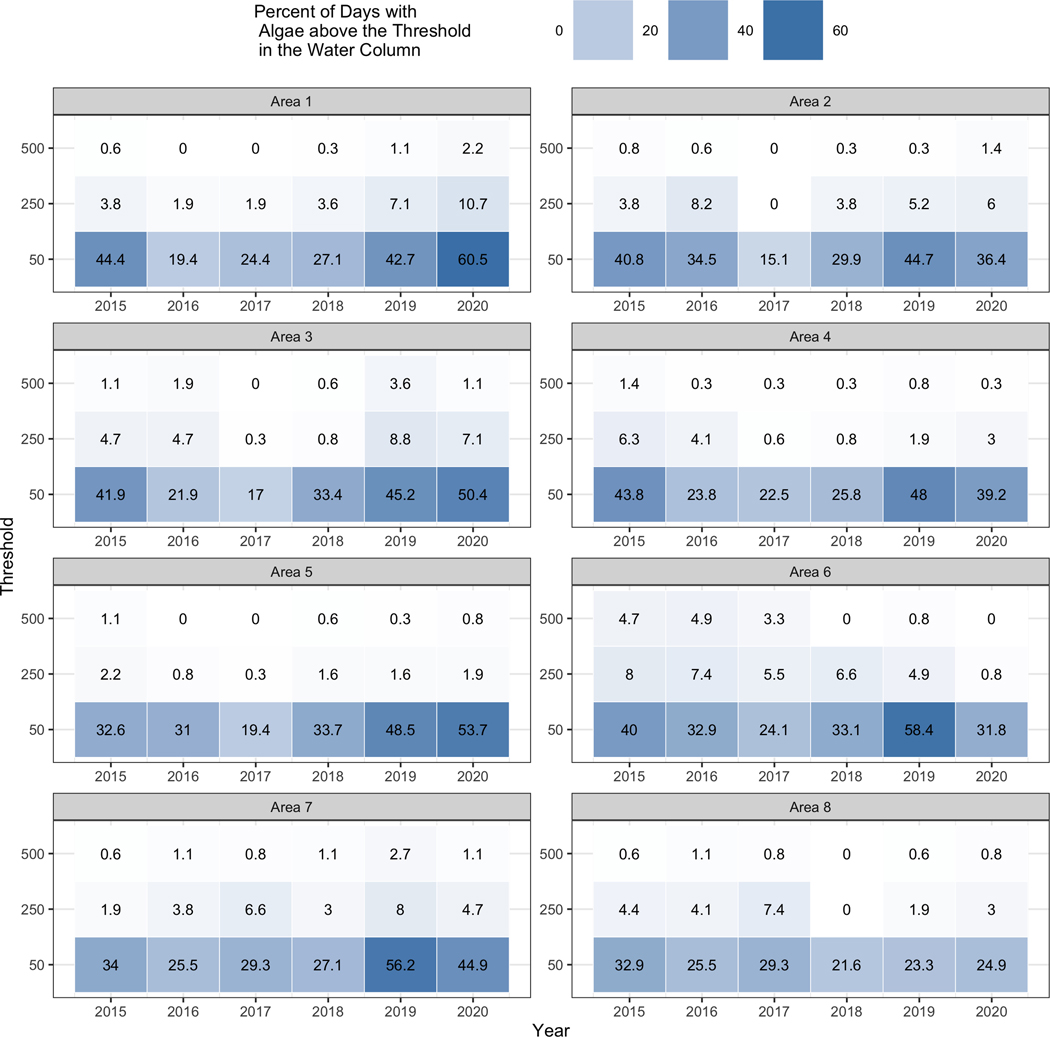
Heat map of predicted percent of days with algae presence in the water column above the three different thresholds. The x axis is years studied and the y axis is the different threshold levels. Each of the eight boxes represents a different area. For this figure, months were averaged over

**Fig. 7:**
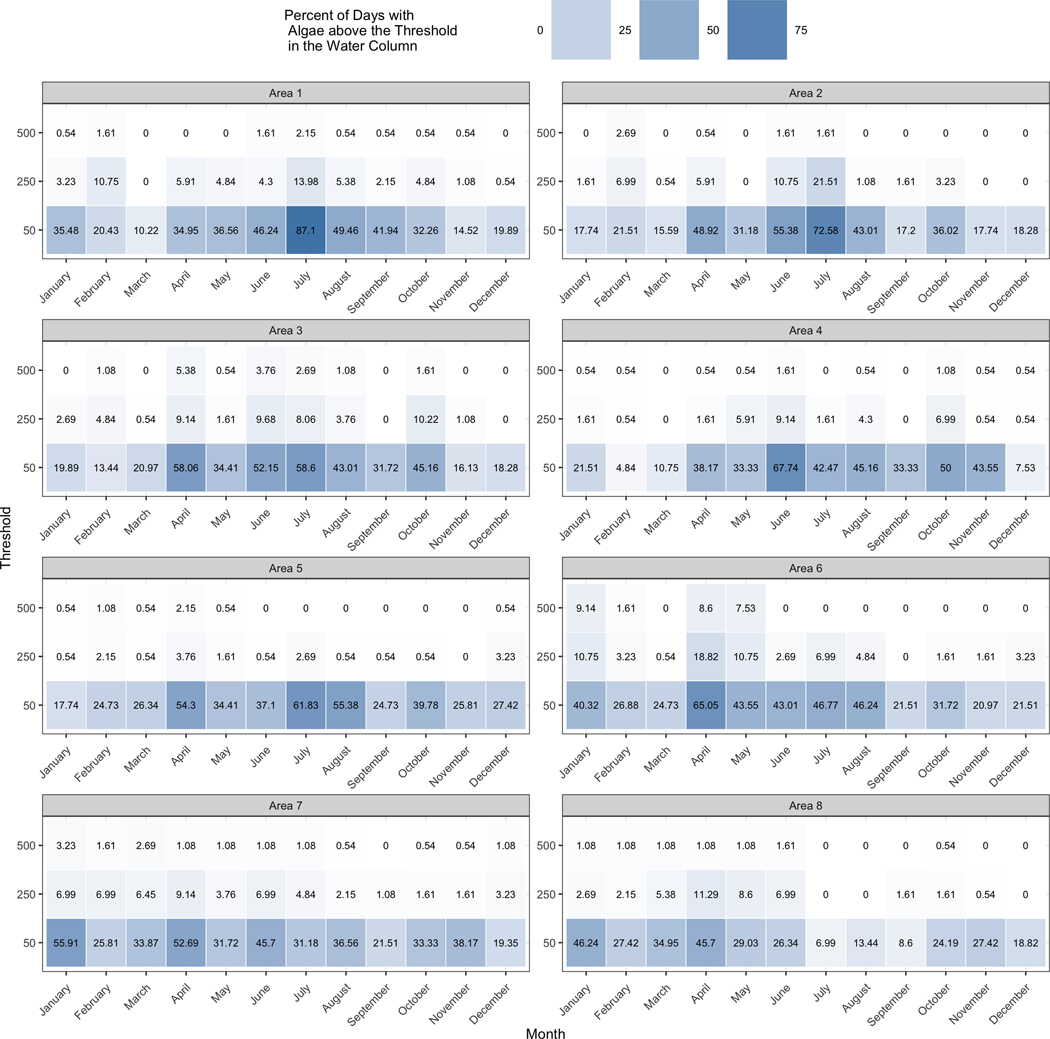
Heat map of predicted percent of days with algae presence in the water column above the three different thresholds. The x axis is months and the y axis is the different threshold levels. Each of the eight boxes represents a different area. For this figure, years were averaged over

**Fig. 8:**
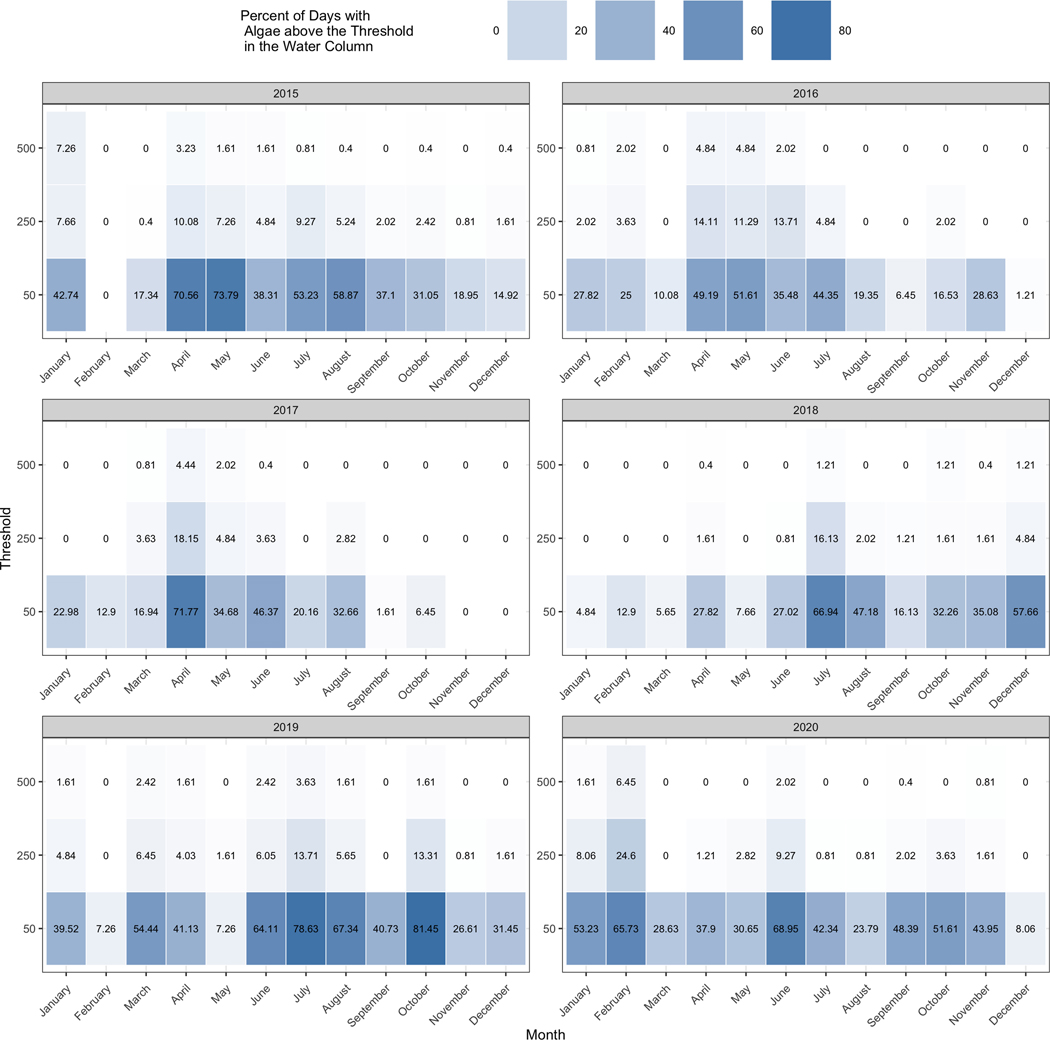
Heat map of predicted percent of days with algae presence in the water column above the three different thresholds. The x axis is months and the y axis is the different threshold levels. Each of the six boxes represents a different year. For this figure, areas were averaged over

**Fig. 9:**
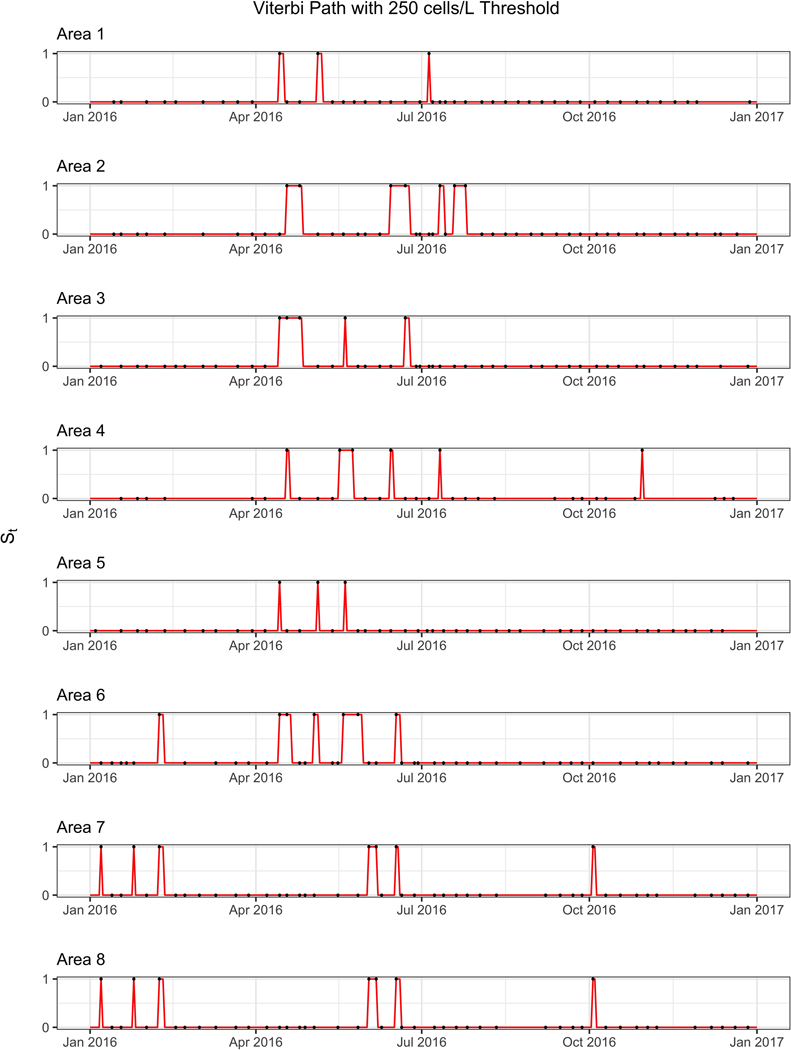
Decoded water column state ($S_{t}$) path using the Viterbi algorithm for 2016 for areas 2–8. The line is the decoded path while the dots indicate absence and presence of observed algae. When algae counts are above the threshold of 250 cells/L, indicated with a black dot at 1, $S_{t}$ must equal one

**Fig. 10:**
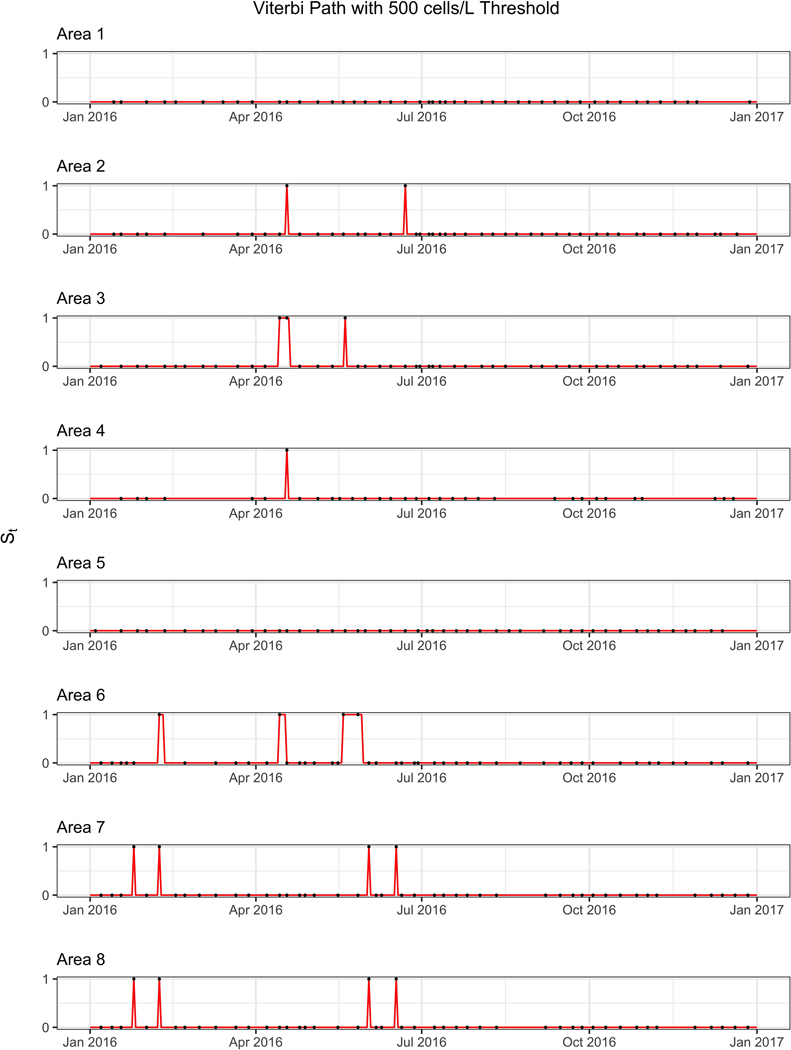
Decoded water column state ($S_{t}$) path using the Viterbi algorithm for 2016 for areas 2–8. The line is the decoded path while the dots indicate absence and presence of observed algae. When algae counts are above the threshold of 500 cells/L, indicated with a black dot at 1, $S_{t}$ must equal one

## Data Availability

The water sample algae count and DST measurements are available from the Consejería de Agricultura, Pesca y Desarrollo Sostenible de la Junta de Andalucía upon reasonable request. *Code*

## Appendix A: Varying the algae threshold

This appendix examines the ramifications of adjusting the algae detection threshold. Noted previously in prior sections, when any amount of algae is present in the water sample we consider algae present in the water column. Although this metric is useful for specific scenarios, such as quantifying chronic exposure, it cannot single out larger events like harmful algae blooms. Because algae is present year-round, when a lower threshold is chosen larger algae events are mixed in with smaller algae events. As this interpretation may not be sufficient for a study of harmful algae blooms, we consider three different thresholds. By raising the threshold for what we consider algae presence to be, we can study larger algae events beyond presence. The two additional thresholds examined are at 250 and 500 cells/L. 500 cells/L was established by the Andalucía HAB monitoring program as a critical threshold, and 250 cells/L was chosen as a halfway point.

As can be seen in the three following heat maps, when the algae threshold is increased our model predicts fewer days with algae in the water column. Thus, by raising the threshold our model is able to be more precise and pick out days with larger algae events. The drastic decrease in predicted algae positive days as the threshold increases indicates that under the original 50 cells/L threshold, most of the algae positive days had a low predicted amount of algae.

Figure 6 shows the predicted percent of days with algae presence in the water column above the three different thresholds, averaged over months, for six years and eight different sites. By summing over months, we can look at yearly trends. When the threshold is 50 cells/L this figure contains the same information as Fig. 5. The U-shaped pattern present at 50 cells/L, where the earlier and later years studied had higher predicted algae presence in the water column when compared to the middle years, is still prevalent for both the 250 and 500 cells/L threshold for areas 1–5. Areas 6 has a higher predicted algae presence above the threshold in the water column in the beginning and then slowly decreases from 2015 to 2020 for both the additional thresholds while area 7 and 8 vary by year.

Figure 7 shows the predicted percent of days with algae presence in the water column above the three different thresholds, averaged over years, for each month and the eight different sites. This heat map focuses on monthly trends as it sums over years. For the 50 cells/L threshold, the number of predicted days where algae is above the threshold in the water column peaks between April and August, often occurring in May. Areas 1–6 all had the highest percentages during the spring and summer months, while areas 7 and 8 also had a high predicted percentage in January. Looking at the 250 cells/L threshold, the highest percentages occurred between April and October, with most peaks happening in April. Areas 1, 2, 6, and 7 also had at least one winter month with a high predicted percentage, however all areas had the most predicted positive days during the spring and summer months. Finally, for the 500 cells/L threshold areas 6 and 7 had the highest predicted percent of days with algae above the threshold in January, however area 6 also has high predictions for April and May. Area 2 has the highest predicted percent of days in February but is closely followed by June and July. The rest of the areas have the highest predicted percent of days between April and July.

Figure 8 shows the predicted percent of days with algae presence in the water column above the three different thresholds, averaged over areas, for each month and year. This heat map sums over geographic differences and lets us examine overall trends, looking at western Andalucía as a whole. Across all years and thresholds (except for the 500 cells/L threshold in 2020), there is a higher predicted percent of days of algae above the threshold in the water column during the spring and summer, although some years had increased algae during the winter as well.

Figures 9 and 10 are similar to Figs. 2 and 3 and show the interpolated Viterbi path for 2016 when the threshold is 250 and 500 cells/L, respectively. Again, as we increase the threshold, the qualification for what counts as algae presence in the water column is harder to meet. Therefore, fewer days are predicted to have algae in the water column, however the predicted algae events are larger. The decrease in number of days that are estimated to have algae in the water column when the threshold is increased can also be seen in these two plots as the Viterbi path in Fig. 9 passes through the 1 state more often than the Viterbi path in Fig. 10 does.

## Abbreviations

OA: Okadaic acid
DST: Diarrhetic shellfish toxins
DSP: Diarrhetic shellfish poison
HMM: Hidden markov models

## References

[R1] Bartolucci F, Bacci S, Pennoni F (2014) Longitudinal analysis of self-reported health status by mixture latent auto-regressive models. J R Stat Soc Ser C Appl Stat. 10.1111/rssc.12030

[R2] Baum LE (1972) An inequality and associated maximization technique in statistical estimation for probabilistic functions of Markov processes. In: Shisha O (Ed) Inequalities III: proceedings of the third symposium on inequalities. University of California, Los Angeles, Academic Press, pp 1–8

[R3] Blanco J, Martín-Morales E, Alvarez G (2018) Stability of okadaic acid and 13-desmethyl spirolide c in seawater and sediment. Marine Chem. 10.1016/j.marchem.2018.10.007

[R4] Bouza N, Aboal M (2008, 01) Fitoplanctón potencialmente tóxico en la costa sur de murcia (so mar mediterráneo). Avances y tendencias en fitoplancton tóxico y biotoxinas, 2008-01-01, ISBN 978-84-96997-06-6, pags. 77–86

[R5] FAO, WHO (2016) Toxicity equivalence factors for marine biotoxins associated with bivalve molluscs. http://www.fao.org/3/a-i5970e.pdf

[R6] Fernández R, Maman L, Jaén D, Fernández Fuentes L, Ocaña García-Donas M, Gordillo M (2019) Dinophysis species and diarrhetic shellfish toxins: 20 years of monitoring program in Andalusia, south of Spain. Toxins 11:189. 10.3390/toxins11040189 30934968 PMC6520784

[R7] Fujiki H, Sueoka E, Watanabe T, Suganuma M (2018) The concept of the okadaic acid class of tumor promoters is revived in endogenous protein inhibitors of protein phosphatase 2a, set and cip2a, in human cancers. J Cancer Res Clin Oncol. 10.1007/s00432-018-2765-7 PMC6244643 30341686

[R8] Fux E, Smith J, Tong M, Guzmán L, Anderson D (2011) Toxin profiles of five geographical isolates of *Dinophysis* spp. from north and south America. Toxicon 57:275–87. 10.1016/j.toxicon.2010.12.002 21147146

[R9] Gao H, An X, Liu L, Zhang K, Zheng D, Tong M (2017) Characterization of dinophysis acuminata from the yellow sea, china, and its response to different temperatures and mesodinium prey. Oceanol Hydrobiol Stud. 10.1515/ohs-2017-0043

[R10] IOC, FAO, WHO (2005) Report of the joint fao/ioc/who ad hoc expert consultation on biotoxins in bivalve molluscs: short summary. UNESCO. https://unesdoc.unesco.org/ark:/48223/pf0000139421

[R11] Jiang P, Liu XJZ, Yuan X (2016) A framework based on hidden Markov model with adaptive weighting for microcystin forecasting and early-warning. Decis Supp Syst. 10.1016/j.dss.2016.02.003

[R12] Juang B, Rabiner L (1986) Mixture autoregressive hidden markov models for speaker independent isolated word recognition. In: ICASSP’86. IEEE international conference on acoustics, speech, and signal processing, vol 11, pp 41–44. 10.1109/ICASSP.1986.1169183

[R13] Juang B-H, Rabiner L (1985) Mixture autoregressive hidden markov models for speech signals. IEEE Trans Acoust Speech Signal Process 33(6):1404–1413. 10.1109/TASSP.1985.1164727

[R14] Kim KB, Jung M-K, Tsang YF, Kwon H-H (2020) Stochastic modeling of chlorophyll-a for probabilistic assessment and monitoring of algae blooms in the lower Nakdong river, South Korea. J Hazard Mater 400:123066. 10.1016/j.jhazmat.2020.123066 32593943

[R15] Kulawiak M (2016) Operational algae bloom detection in the Baltic sea using Gis and Avhrr data. Baltica 29:3–18. 10.5200/baltica.2016.29.02

[R16] Mafra L, Schramm M, Tavares C (2013) Diarrheic toxins in field-sampled and cultivated dinophysis spp. cells from southern brazil. J Appl Phycol. 10.1007/s10811-013-0219-9

[R17] Pizarro G, Paz B, González-Gil S, Franco MJ, Reguera B (2009) Seasonal variability of lipophilic toxins during a dinophysis acuta bloom in western iberia: differences between picked cells and plankton concentrates. Harmful Algae 8(6):926–937. 10.1016/j.hal.2009.05.004

[R18] Rabiner L, Juang B (1986) An introduction to hidden Markov models. IEEE ASSP Mag 3(1):4–16. 10.1109/MASSP.1986.1165342

[R19] Reguera B, Riobó P, Rodríguez F, Díaz P, Pizarro G, Paz B, Blanco J (2014) Dinophysis toxins:causative organisms, distribution and fate in shellfish. Mar Drugs 12:394–461. 10.3390/md12010394 24447996 PMC3917280

[R20] Reguera B, Velo-Suárez L, Raine R, Gil Park M (2012) Harmful dinophysis species: a review. Harmful Algae 14:87–106. 10.1016/j.hal.2011.10.016

[R21] Rousseeuw K, Poison Caillault E, Lefebvre A, Hamad D (2015) Hybrid hidden Markov model for marine environment monitoring. IEEE J Select Top Appl Earth Observ Remote Sens 8(1):204–213. 10.1109/JSTARS.2014.2341219

[R22] Shannon M, Byrne W (2010, 09) Autoregressive hmms for speech synthesis

[R23] Stanculescu I, Williams C, Freer Y (2014) Autoregressive hidden Markov models for the early detection of neonatal sepsis. IEEE J Biomed Health Inform 18:1560–1570. 10.1109/JBHI.2013.2294692 25192568

[R24] Suganuma M, Fujiki H, Suguri H, Yoshizawa S, Hirota M, Nakayasu M, Sugimura T (1988) Okadaic acid: an additional non-phorbol-12-tetradecanoate-13-acetate-type tumor promoter. Proc Natl Acad Sci 85(6):1768–1771. 10.1073/pnas.85.6.1768 3126494 PMC279860

[R25] Urban P, Rezaei Tabar V, Denkiewicz M, Bokota G, Das N, Basu S, Plewczynski D (2020) The mixture of autoregressive hidden Markov models of morphology for dentritic spines during activation process. J Comput Biol. 10.1089/cmb.2019.0383 PMC7482113 32175768

[R26] Valdiglesias V, Prego-Faraldo M, Pásaro E, Mendez J, Laffon B (2013) Okadaic acid: more than a diarrheic toxin. Mar Drugs 11:4328–49. 10.3390/md11114328 24184795 PMC3853731

[R27] Velo-Suárez L, Estrada J (2007) Artificial neural network approaches to one-step weekly prediction of *Dinophysis acuminata* blooms in Huelva (western Andalucía, Spain). Harmful Algae 6:361–371. 10.1016/j.hal.2006.11.002

[R28] Yasumoto T, Murata M, Oshima Y, Matsumoto GK, Clardy J (1984) Diarrhetic shellfish poisoning. In: Seafood toxins, pp 207–214. 10.1021/bk-1984-0262.ch019

[R29] Yasumoto T, Oshima Y, Sugawara W, Fukuyo Y, Oguri H, Igarashi T, Fujita N (1980) Identification of *Dinophysis fortii* as the causative organism of diarrhetic shellfish poisoning. Nippon Suisan Gakkaishi 46(11):1405–1411. 10.2331/suisan.46.1405

[R30] Yasumoto T, Oshima Y, Yamaguchi M (1978) Occurrence of a new type of shellfish poisoning in the Tohoku district. Nippon Suisan Gakkaishi 44(11):1249–1255. 10.2331/suisan.44.1249

[R31] Zonas de producción. (n.d.). Junta de Andalucia. http://www.juntadeandalucia.es/agriculturaypesca/moluzonasprodu/ZonaProduccionAction.do?accion=filtrarEspecie&accionVC=ok&especie=-1&url=http%3A%2F%2Fwww.juntadeandalucia.es%2Fagriculturaypesca%2Fmoluzonasprodu%2F

[R32] Zucchini W, Macdonald I (2009) Hidden Markov models for time series: an introduction using r. 10.1201/9781420010893

