# Supplementary material for: Modeling Dinophysis in Western Andalucía using an autoregressive hidden Markov model
Source: Environ Ecol Stat. Author manuscript; Available in PMC 2022 Dec 19. (PMC9762684; doi:10.1007/s10651-022-00534-7)
Supplement: Appendix [file NIHMS1819074-supplement-Appendix.pdf]

**Acknowledgements** We wish to acknowledge the Consejería de Agricultura, Pesca y Desarrollo Sostenible de la Junta de Andalucía for discussions and the study data.The authors would also like to acknowledge discussions with Clarissa Anderson, executive director of the southern California coastal ocean observing system at Scripps oceanography. This work utilized the computational resources of the NIH HPC Biowulf cluster. Dr. Gribble's effort was supported in part by funding from the National Institute of Environmental Health Sciences (P30ES019776 and R01ES029165).

**Data Availibility Statement** The water sample algae count and DST measurements are available from the Consejería de Agricultura, Pesca y Desarrollo Sostenible de la Junta de Andalucía upon reasonable request.

**Code** Code is available at https://github.com/jordanaron22/AndalusiaAlgae

## Declarations

**Conlict of Interest** None declared.

**Open Access** This article is licensed under a Creative Commons Attribution 4.0 International License, which permits use, sharing, adaptation, distribution and reproduction in any medium or format, as long as you give appropriate credit to the original author(s) and the source, provide a link to the Creative Commons licence, and indicate if changes were made. The images or other third party material in this article are included in the article's Creative Commons licence, unless indicated otherwise in a credit line to the material. If material is not included in the article's Creative Commons licence and your intended use is not permitted by statutory regulation or exceeds the permitted use, you will need to obtain permission directly from the copyright holder. To view a copy of this licence, visit http://creativecommons.org/licenses/by/4.0/.

## Appendix A: Varying the algae threshold

This appendix examines the ramifications of adjusting the algae detection threshold. Noted previously in prior sections, when any amount of algae is present in the water sample we consider algae present in the water column. Although this metric is useful for specific scenarios, such as quantifying chronic exposure, it cannot single out larger events like harmful algae blooms. Because algae is present year-round, when a lower threshold is chosen larger algae events are mixed in with smaller algae events. As this interpretation may not be sufficient for a study of harmful algae blooms, we consider three different thresholds. By raising the threshold for what we consider algae presence to be, we can study larger algae events beyond presence. The two additional thresholds examined are at 250 and 500 cells/L. 500 cells/L was established by the Andalucía HAB monitoring program as a critical threshold, and 250 cells/L was chosen as a halfway point.

As can be seen in the three following heat maps, when the algae threshold is increased our model predicts fewer days with algae in the water column. Thus, by raising the threshold our model is able to be more precise and pick out days with larger algae events. The drastic decrease in predicted algae positive days as the threshold increases indicates that under the original 50 cells/L threshold, most of the algae positive days had a low predicted amount of algae.

Figure 6 shows the predicted percent of days with algae presence in the water column above the three different thresholds, averaged over months, for six years and eight different sites. By summing over months, we can look at yearly trends. When the threshold is 50 cells/L this figure contains the same information as Fig. 5. The

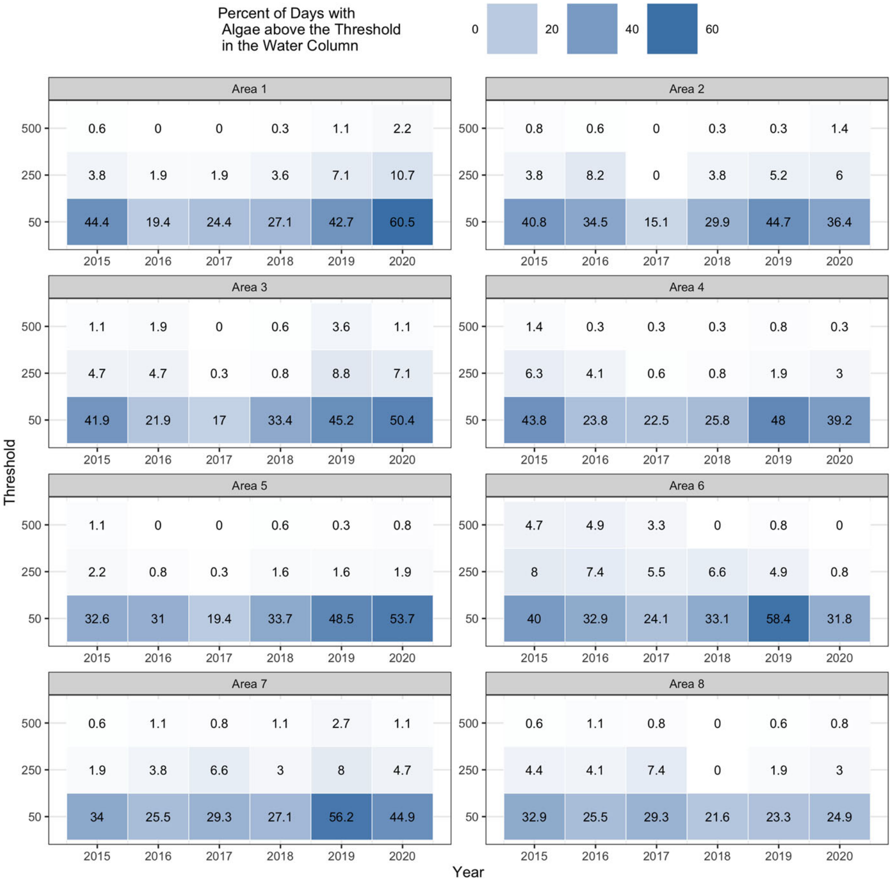


**Fig. 6** Heat map of predicted percent of days with algae presence in the water column above the three different thresholds. The x axis is years studied and the y axis is the different threshold levels. Each of the eight boxes represents a different area. For this figure, months were averaged over

U-shaped pattern present at 50 cells/L, where the earlier and later years studied had higher predicted algae presence in the water column when compared to the middle years, is still prevalent for both the 250 and 500 cells/L threshold for areas 1-5. Areas 6 has a higher predicted algae presence above the threshold in the water column in the beginning and then slowly decreases from 2015 to 2020 for both the additional thresholds while area 7 and 8 vary by year.

Figure 7 shows the predicted percent of days with algae presence in the water column above the three different thresholds, averaged over years, for each month and the eight different sites. This heat map focuses on monthly trends as it sums over years. For the 50 cells/L threshold, the number of predicted days where algae is above the threshold in the water column peaks between April and August, often occurring in May. Areas 1-6 all had the highest percentages during the spring and summer months, while areas 7 and 8 also had a high predicted percentage in January. Looking at the 250

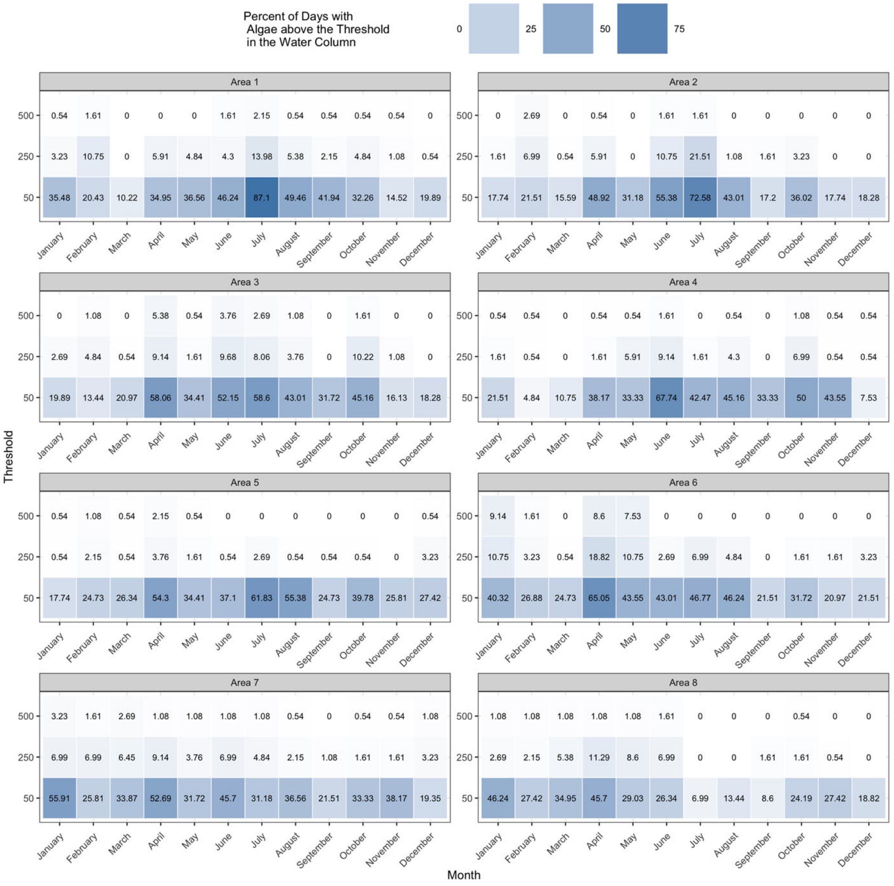


**Fig. 7** Heat map of predicted percent of days with algae presence in the water column above the three different thresholds. The x axis is months and the y axis is the different threshold levels. Each of the eight boxes represents a different area. For this figure, years were averaged over

cells/L threshold, the highest percentages occurred between April and October, with most peaks happening in April. Areas 1, 2, 6, and 7 also had at least one winter month with a high predicted percentage, however all areas had the most predicted positive days during the spring and summer months. Finally, for the 500 cells/L threshold areas 6 and 7 had the highest predicted percent of days with algae above the threshold in January, however area 6 also has high predictions for April and May. Area 2 has the highest predicted percent of days in February but is closely followed by June and July. The rest of the areas have the highest predicted percent of days between April and July.

Figure 8 shows the predicted percent of days with algae presence in the water column above the three different thresholds, averaged over areas, for each month and year. This heat map sums over geographic differences and lets us examine overall trends, looking at western Andalucía as a whole. Across all years and thresholds (except for

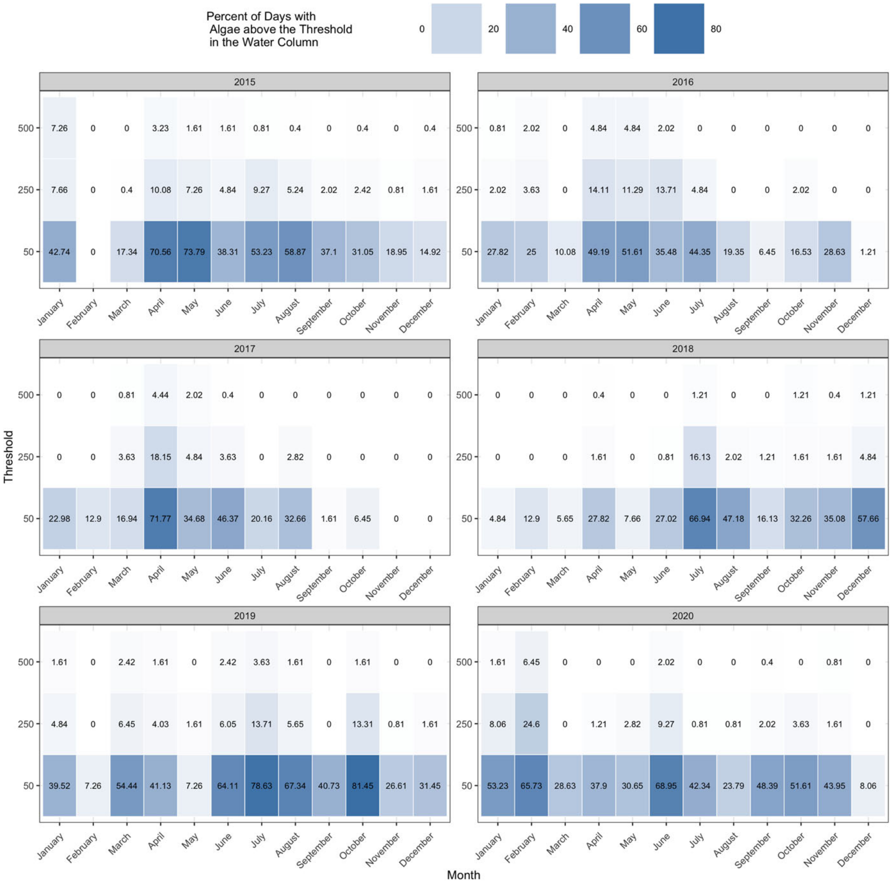


**Fig. 8** Heat map of predicted percent of days with algae presence in the water column above the three different thresholds. The x axis is months and the y axis is the different threshold levels. Each of the six boxes represents a different year. For this figure, areas were averaged over

the 500 cells/L threshold in 2020), there is a higher predicted percent of days of algae above the threshold in the water column during the spring and summer, although some years had increased algae during the winter as well.

Figures 9 and 10 are similar to Figs. 2 and 3 and show the interpolated Viterbi path for 2016 when the threshold is 250 and 500 cells/L, respectively. Again, as we increase the threshold, the qualification for what counts as algae presence in the water column is harder to meet. Therefore, fewer days are predicted to have algae in the water column, however the predicted algae events are larger. The decrease in number of days that are estimated to have algae in the water column when the threshold is increased can also be seen in these two plots as the Viterbi path in Fig. 9 passes through the 1 state more often than the Viterbi path in Fig. 10 does.
